# Stable platelet production via the bypass pathway explains long-term hematopoietic stem cell reconstitution

**DOI:** 10.1016/j.isci.2025.112547

**Published:** 2025-04-29

**Authors:** Shoya Iwanami, Toshiko Sato, Hiroshi Haeno, Longchen Xu, Keimyo Imamura, Jun Ooehara, Xun Lan, Hiromitsu Nakauchi, Shingo Iwami, Ryo Yamamoto

**Affiliations:** 1 interdsciplinary Biology Laboratory (iBLab), Division of Natural Science, Graduate School of Science, Nagoya University, Nagoya, Japan; 2 Institute for the Advanced Study of Human Biology (ASHBi), Institute of Advanced Study, Kyoto University, Kyoto, Japan; 3 Research Institute for Biomedical Science, Tokyo University of Science, Noda, Japan; 4 Tsinghua-Peking Center for Life Sciences, Tsinghua University, Beijing, China; 5 Department of Basic Medical Sciences, School of Medicine, Tsinghua University, Beijing, China; 6 Institute for Stem Cell Biology and Regenerative Medicine, Stanford Medicine, Stanford, CA 94305, USA; 7 International Research Center for Neurointelligence, The University of Tokyo Institutes for Advanced Study, The University of Tokyo, Tokyo, Japan; 8 Institute of Mathematics for Industry, Kyushu University, Fukuoka, Japan; 9 Interdisciplinary Theoretical and Mathematical Sciences Program (iTHEMS), RIKEN, Saitama, Japan; 10 NEXT-Ganken Program, Japanese Foundation for Cancer Research (JFCR), Tokyo, Japan; 11 Science Groove Inc., Fukuoka, Japan; 12 Stem Cell Therapy Division, Institute of Integrated Research, Institute of Science, Tokyo, Japan

**Keywords:** Stem cells research, Mathematical biosciences, Biological constraints

## Abstract

*In vivo* differentiation pathways into several blood cell lineages of Hematopoietic stem cells (HSCs) remain challenging to track over time. Using data from single-cell transplantation assays and mathematical modeling, we examined HSC differentiation kinetics, including the myeloid bypass pathway. We found that myeloid cell production was unchanged with age, whereas B cell production declined, quantitatively confirming myeloid lineage skewing. Estimated dependence on the platelet-bypass correlated with the long-term reconstitution capacity of HSCs. Time-dependent blood cell production patterns calculated by our model distinguished the reconstitution potential of HSCs into subgroups, suggesting a link between the bypass pathway and the multilineage differentiation dynamics of HSCs. Notably, platelet bypass dependence could be determined by the platelet-to-erythrocyte chimerism ratio at 8 weeks after transplantation, serving as a predictive indicator of long-term HSC function. These findings provide quantitative insights into HSC aging and differentiation dynamics, emphasizing the role of the bypass pathway in defining HSC properties.

## Highlights

• The differentiation of single-HSC clones was analyzed in a mathematical model
• Dependence on the platelet bypass correlates with long-term HSC reconstitution
• Blood cell production patterns distinguished the reconstitution potential of HSCs
• Early platelet-to-erythrocyte chimerism ratio predicted HSC characteristics

## Graphical abstract

**Figure:**
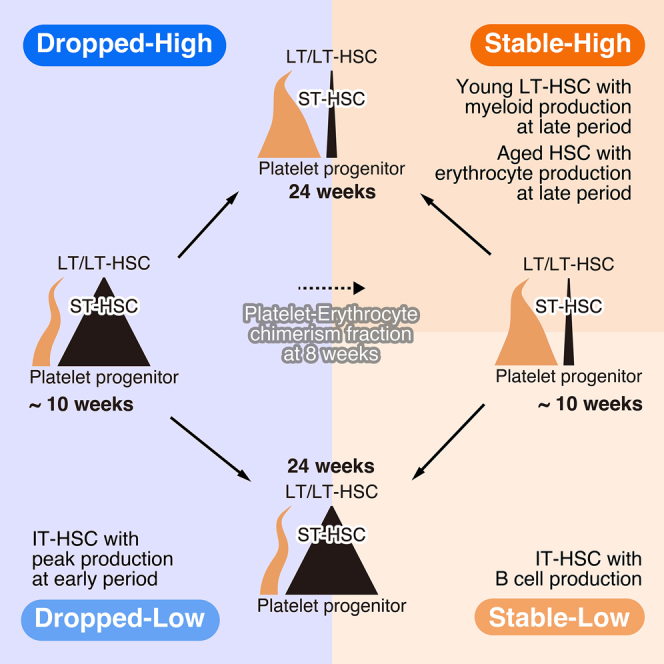

## Introduction

Although it is known that mouse hematopoietic stem cells (HSCs) are found within the phenotypic CD150^+^CD34^-/low^Flt3^−^c-Kit^+^Sca-1^+^Lineage^−^ (CD150^+^CD34^−^KSL) cell population,^1^^,^^2^ we are still unable to prospectively identify functional HSCs with 100% accuracy.^3^ One powerful tool for characterizing the heterogeneity of HSCs is the single-cell transplantation assay. This tool evaluates the functional properties of transplanted cells by the duration of hematopoietic reconstitution and the blood cell lineages produced in the primary and secondary transplantations. In our previous studies using single-cell transplantation, we showed that HSCs producing all blood cell lineages can be distinguished with regard to the duration of reconstitution as long-term (LT-), intermediate-term (IT-), and short-term (ST-) HSCs.^4^^,^^5^ Although single-cell transplantation experiments reveal the nature of the transplanted cells, this information is gained retrospectively, and long-term observation is required to determine cell function.

We have previously shown using clonal transplantation assays that single HSCs demonstrate self-renewal and multipotency.^6^ Furthermore, in the phenotypically defined HSC compartment, we found long-term repopulating cells with differentiation potential restricted to the myeloid lineage at a single-cell level. These myeloid-restricted stem cells (MySCs) include three subtypes: common myeloid stem cells (CMSCs), which generate megakaryocytes, erythrocytes, and myeloid cells; megakaryocyte-erythroid stem cells (MESCs), which produce megakaryocytes and erythrocytes; and megakaryocyte stem cells (MkSCs), which solely generate megakaryocytes.^4^^,^^7^ Additionally, we identified a change in the frequency of MySCs with aging.^5^ Paired-daughter cell assay combined with single-cell transplantation demonstrated the possible existence of a myeloid bypass pathway from HSCs; however, this assay was partially performed *in vitro*. Therefore, we cannot deny the possibility that this bypass pathway is an artificial phenomenon *in vitro*.

Analysis of HSC populations primed for platelet-specific gene expression in similar transplantation assays has shown a relationship between platelet-biased blood cell production and the long-term reconstitution capacity of hematopoiesis.^8^ Other studies using single-cell transplantation assays have observed limited patterns in the production of blood cells into multiple lineages from HSCs, particularly cells with long-term reconstitution capacity and platelet lineage-restricted production.^5^^,^^9^ These findings suggest a connection between the presence of the bypass pathway and the differentiation potential of HSCs. Recently, a nonhierarchical relationship between HSCs and platelet-biased stem cells and their two distinct pathways for platelet production have been demonstrated.^10^ How the bypass pathway works in the *in vivo* hematopoietic process has important implications for our understanding of the entire hematopoiesis.

We hypothesized that mathematical modeling of the chimerism observed in peripheral blood (PB) could help to determine how hematopoiesis from HSCs depends on myeloid bypass using *in vivo* data alone. Using mathematical models to estimate cell differentiation kinetics allows us to understand the phenomena underlying time-related changes. For example, in a study analyzing the time course of leukemia cells in patients with chronic myeloid leukemia, investigators used the estimated decay rate of leukemic cells to identify target cells for therapeutic drugs and elucidate the mechanism of reactivation due to the emergence of resistant mutations after treatment.^11^ In the multilineage differentiation of HSCs, mathematical models have been used to quantify the cell cycle changes associated with cell divisions and the subsequent bias in differentiation lineages.^12^ Mathematical models have also been used to estimate differentiation pathways, which enabled comparison of the contribution of multiple pathways to blood cell production.^13^ Theoretical analysis of the competitive proliferation of cells in transplantation assays and the reconstitution capacity of HSCs has also been conducted by use of a hierarchical cell differentiation model.^14^

Here, we sought to dissect LT-, IT- and ST-HSCs in the transplantation setting based on the reconstitution kinetics (chimerism) of the four lineages of blood cells (neutrophils/monocytes, erythrocytes, platelets, and B cells) using a mathematical model and to investigate how LT-, and IT-HSCs produce mature blood cell lineages via the myeloid bypass pathway. By applying mathematical modeling and clustering methodologies to functional chimerism datasets generated from 75 single-cell transplantation assays, we have proposed a classification of four LT- and IT-HSC subsets. We found that HSCs biased toward platelets compared with erythrocytes at an early point after transplantation tended to be of the stable bypass subsets. Our large-scale datasets therefore provide a useful “atlas” of LT- and IT-HSC heterogeneity.

## Results

### Evaluation of blood cell production by hematopoietic reconstitution in transplantation assays and time series data

**Figure 1:**
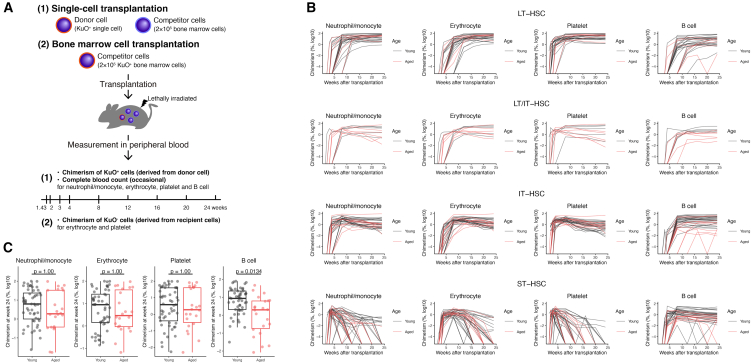
Evaluation of hematopoietic reconstitution capacity by transplantation assays of hematopoietic cells (A) Schematic representation of the single-cell transplantation assay of pHSCs and bone marrow cells. In the single-cell transplantation assay, single pHSCs (donor cells) obtained from donor mice expressing Kusabira-Orange (KuO) were transplanted into lethally irradiated recipient mice along with $2\times 10^{5}$ bone marrow cells (competitor cells). The percentage of cells derived from donor mouse cells in each blood cell lineage in the PB (chimerism) was measured over 24 weeks. In part of the transplantation assays, complete blood cell count (CBC) was measured at the same time. Details of the transplantation assays and data collection were described in previous studies.^4^^,^^5^ In the KuO^+^ bone marrow cell transplantation assays, the percentage of KuO^−^ cells in the peripheral blood up to 8 weeks after transplantation was measured as chimerism to assess recipient cell decay. (B) The time-course change in chimerism obtained in single-cell transplantation assays for each class, LT-HSCs, LT/IT-HSCs, IT-HSCs, and ST-HSCs, defined by reconstitution capacity. The black and red lines indicate chimerism of single cells obtained from young and aged mice, respectively. Data later in discussion the detection limit are displayed with chimerism as 0.001%. (C) Comparison of chimerism from LT-HSCs, LT/IT-HSCs, and IT-HSCs for each blood cell lineage at 24 weeks after transplantation in the single-cell transplantation assay with aging. P-values were calculated by the Student’s t test for the mean of young and aged HSCs with Bonferroni correction and a significance level as 0.05.

In the transplantation assays used to evaluate the characteristics and capacity of cells involved in hematopoiesis, cells from labeled donor mice are transplanted into a recipient mouse. Then, the fraction, or chimerism, of cells in PB derived from the transplanted single cells (donor cells) is measured as functional blood cell production (Figure 1A). In this study, we analyzed data from single-cell transplantation assays of phenotypic HSCs (pHSCs) performed in previous studies^4^^,^^5^^,^^15^ with the aim of elucidating the differentiation kinetics of HSCs. Chimerism values for neutrophils/monocytes, erythrocytes, platelets, and B cells from a total of 114 recipient mice were used (Table 1). Of the 114 mice, 81 were transplanted with young HSCs (8–12 weeks of age) and the remaining 33 were transplanted with aged HSCs (20–24 months of age). The class of transplanted single cells was defined according to the duration of hematopoietic reconstitution, with chimerism values measured according to a defined threshold during primary and secondary transplantation. A total of 29, 35, and 39 single cells were classified as long-term HSCs (LT-HSCs), intermediate-term HSCs (IT-HSCs), and short-term (ST-HSCs), respectively. The remaining 11 single cells were not evaluated by secondary transplantation and could not be distinguished as LT-HSC or IT-HSC (i.e., LT/IT-HSCs). Here, we used the chimerism data from the first transplantation only, so that LT-HSCs, LT/IT-HSCs, and IT-HSCs were considered as a single population named LT-HSCs in our mathematical model for the following analysis. Complete blood cell count (CBC) was measured in 30 of the transplantations for the experiments that fit the criteria for data use (STAR Methods). It is difficult to compare time changes in chimerism between young and aged HSCs in single-cell transplantation assays, partly because each class of HSCs is arbitrarily classified based on chimerism data (Figure 1B). Comparing LT-HSC, LT/IT-HSC, and IT-HSC chimerism at week 24 between young and aged mice, there was a significant trend toward a decrease with age in B cells only (*p*-value was 0.0134, Figure 1C). As for the definition of HSC classes, there were clear differences in chimerism between LT-, IT/LT-, or IT-HSC and ST-HSC, but the differences between LT-, IT/LT-, and IT-HSC were unclear, and the chimerisms were highly heterogeneous (Figure 1B). In addition to the above transplantation assays, KuO^+^ bone marrow cells were newly transplanted into 10 mice to obtain time-course data to quantify the decay in erythrocytes and platelets in the PB of the KuO^−^ recipient mice (Table 1, Figure S3 and STAR Methods). See Table 1, STAR Methods, and the original studies^4^^,^^5^ for details on the experiment and the dataset.

**Table 1:** Summary of the number of transplantation assays used for data analysis

Transplanted KuO^+^ cells	Single cells (*N* = 114 recipient mice)				Bone marrow cells (*N* = 10 mice)
Data type	Class				Recipient
	LT-HSC in modeling (LT-HSC:LT/IT-HSC:IT-HSC)		ST-HSC		
	Young	Old	Young	Old	
Chimerism	53 (23:5:25)	22 (6:6:10)	28	11	10
CBC	10 (3:1:6)	7 (3:1:3)	6	7	–

### A mathematical model of the direct myeloid lineage production pathway from hematopoietic stem cells explains blood cell production *in vivo*

**Figure 2:**
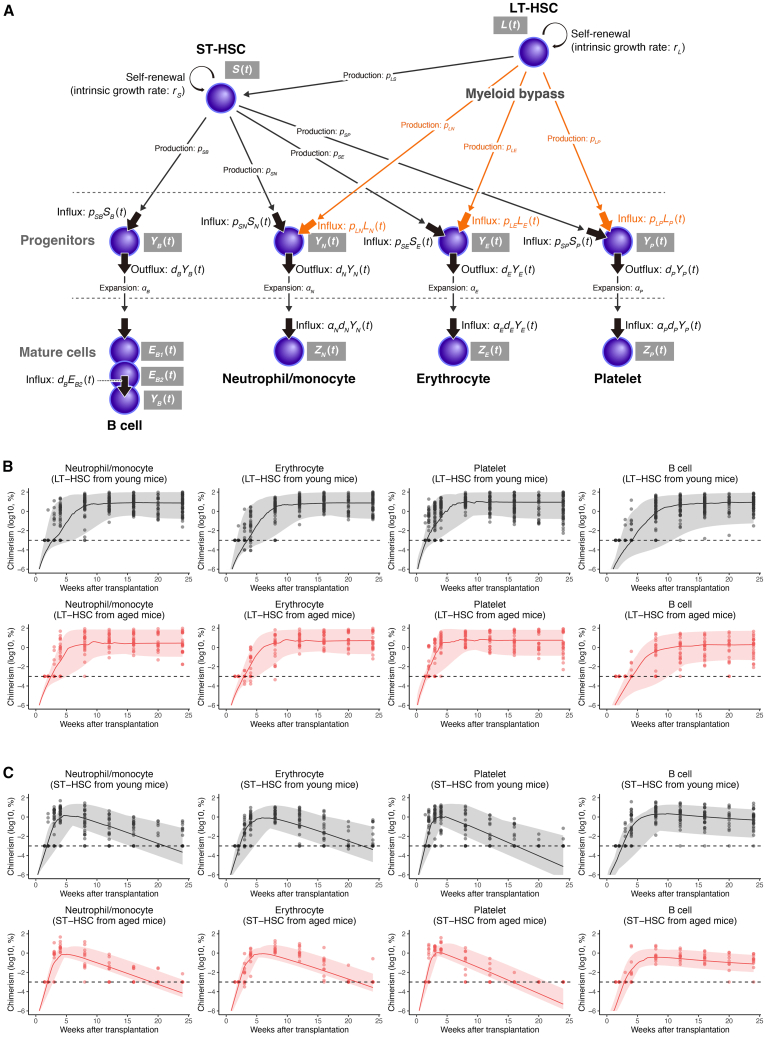
Summary of the data fitting to chimerism data using a mathematical model (A) Overview of the hierarchical structure of the differentiation model. The pathway for differentiation from LT-HSCs to progenitor cells that does not go through ST-HSCs is termed the *myeloid bypass pathway* and is indicated by the red arrows. Proliferation by self-renewal and blood cell production by the differentiation of each cell population from LT-HSCs to ST-HSCs and from LT-HSCs or ST-HSCs to mature cells via progenitor cells, as described in the mathematical model. The number of cells flowing into the lower cell population as a result of cell differentiation from the cell population in the upper layers is defined as *influx*. (B and C) Value of chimerism (dots) and predictions calculated by the mathematical model in transplantation assays of single cells obtained from young **(B)** and aged **(C)** mice. The solid lines show the median of the mathematical model’s predictions and the filled area represents the minimum and maximum of the chimerism data calculated with estimated individual parameters. See STAR Methods for the calculation of prediction intervals.

The fraction of cells in the PB observed in a single-cell transplantation assay reflects not only the capacity of a single donor cell but also competition with simultaneously transplanted competitor cells and the recipient mouse’s own cells. Chimerism must be evaluated throughout the time course, taking into account the context in which the data are collected. We used a mathematical model describing the hierarchical differentiation of HSCs using ordinary differential equations ((Equation 9), (Equation 10), (Equation 11), (Equation 12), (Equation 13), (Equation 14), (Equation 15)) to capture the time-course changes in data from the single-cell transplantation assays (Figure 2A). Data fitting and numerical simulation with this model were performed to quantitatively understand the capacity of transplanted single cells and the factors contributing to changes in chimerism.^16^ Our model describes blood cell production by the hierarchical differentiation of self-renewing LT-HSCs (i.e., LT-HSCs, LT/IT-HSCs, and IT-HSCs) and ST-HSCs (Figure 2A). In addition, we assumed that there was a differentiation pathway from LT-HSCs to myeloid progenitors that was not via ST-HSCs. This pathway is defined as the *myeloid bypass pathway*, as revealed in previous studies by MySCs, whose differentiation potential is restricted to myeloid cells.^4^^,^^7^ The chimerism of each lineage in the transplantation assay is assumed to be based on the number of mature cells produced from progenitor cells ((Equation 16), (Equation 17)). The amount of each cell population produced at each time point from the upper population is defined as *influx*, which is an indicator of the differentiation potential of HSCs (Figure 2A). In particular, the degree of dependence on the myeloid bypass pathway can be assessed by comparing the influx to myeloid progenitor cells from the two pathways described in the mathematical model. See the STAR Methods for more details on the mathematical model.

To assess the capacity of transplanted single HSCs, we estimated the parameters of the mathematical model by using the chimerism and CBC of neutrophils/monocytes, erythrocytes, platelets, and B cells in the single-cell transplantation assay (Figures 2, S1 and S2). The percentage of cells on the recipient side with the transplantation of bone marrow cells labeled with Kusabira-Orange (KuO) (Figure S3) was obtained independently for this study and used for data analysis. All data were simultaneously used to estimate the parameters of the mathematical model with a nonlinear mixed-effects model. The values of the model parameters estimated as population parameters and individual parameters are listed in Tables S1 and S2, respectively. The chimerism calculated from the estimated individual parameters captured well the early increase of chimerism data in platelets and the late increase in B cells for LT-, LT/IT-, and IT-HSCs (Figures 2B and S1A). The reduction of myelocyte lineage chimerism and sustained production of B cells in ST-HSCs were also well represented by the mathematical model (Figures 2C and S1B). The predictions computed by the mathematical model were able to explain the overall trends in the complex relationships among the time-varying of data for blood cell lineages in the transplantation assays (Figure S4).

### Age-related changes in hematopoietic stem cell capacity cause a myeloid shift due to a decrease in B cell production

**Figure 3:**
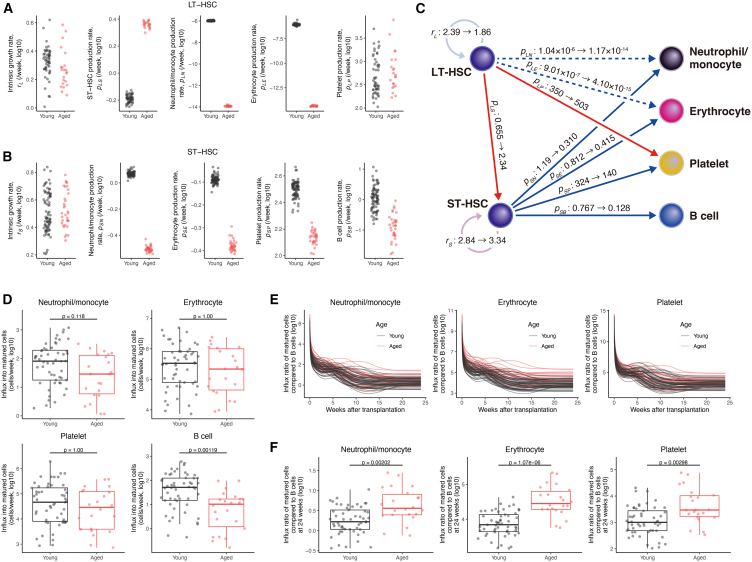
Age-related changes in estimated HSC capacity (A) Age-related changes in the estimated intrinsic growth rate, $r_{L}$, ST-HSC production rate, $p_{LS}$, platelet production rate, $p_{LP}$, erythrocyte production rate, $p_{LE}$, and neutrophil/monocyte production rate, $p_{\mathrm{LN}}$, of LT-HSCs. (B) Age-related changes in the estimated intrinsic growth rate, $r_{S}$, B cell production rate, $p_{SB}$, platelet production rate, $p_{SP}$, erythrocyte production rate, $p_{SE}$, and neutrophil/monocyte production rate, $p_{SN}$, of LT-HSCs. (C) Graphical summary of age-related changes in HSC capacity corresponding to (A and **B)**. Red and blue arrows indicate increases and decreases, respectively. The intensity of the color of the arrow indicates the magnitude of the changes. Few progenitor cells are produced in the pathway depicted by the dashed line. (D) Age-related changes in estimated production (i.e., influx) from progenitors into mature cells in each cell lineage at 24 weeks after transplantation (i.e., $\alpha_{i}d_{i}Y_{i,D,k}\left(24\right)$ (i=N,E,P and $k=n_{L1},...,n_{L75}$) and $d_{B}E_{B2,D,k}\left(24\right)$ ($k=n_{L1},...,n_{L75}$)). (E and F) Production of each lineage of myeloid cells relative to the production of B cells at each time after transplantation (i.e., $\alpha_{i}d_{i}Y_{i,D,k}\left(t\right)/d_{B}E_{B2,D,k}\left(t\right)$ (i=N,E,P and $k=n_{L1},...,n_{L75}$), **(E)**) and at 24 weeks after transplantation (i.e., $\alpha_{i}d_{i}Y_{i,D,k}\left(24\right)/d_{B}E_{B2,D,k}\left(24\right)$ (i=N,E,P and $k=n_{L1},...,n_{L75}$), **(F)**). P-values in **(D)** and **(F)** were calculated by the Student’s t test for the mean of young and aged HSCs with Bonferroni correction and significance level as 0.05.

The values of model parameters for individual single cells estimated from the data fitting allowed us to assess age-related changes in HSC capacity. In our mathematical model, the self-renewal capacity of HSCs corresponds to the intrinsic growth rate of LT-HSCs and ST-HSCs, $r_{L}$ and $r_{S}$, respectively. Comparison of the values of these parameters indicated that the self-renewal capacity of LT-HSCs remained the same or slightly decreased with age (the self-renewal capacity of aged LT-HSCs, 1.86 as population parameter, was 0.780 times that of young LT-HSCs, 2.39, *p*-value was $6.64\times 10^{-2}$ by Wald test, Table S1 and Figure 3A), whereas that of ST-HSCs did not change (the self-renewal capacity of aged ST-HSCs, 3.34 as population parameter, was 1.18 times that of young ST-HSCs, 2.84, *p*-value was 0.217 by Wald test, Table S1 and Figure 3B). The carrying capacities of LT- and ST-HSCs, $L_{s}$ and $S_{s}$, were estimated to be $1.07\times 10^{7}$ and $3.34\times 10^{7}$, respectively, and these values were larger than the HSC population sizes reported in previous studies.^17^^,^^18^ We note that the HSC population that our model assumes is not the same as the populations mentioned in the past studies. These estimates can be interpreted as the ability of HSCs to expand through proliferation relative to the fixed initial values, for LT- or ST-HSCs, $L_{D}\left(0\right)=1/L_{s}$ or $S_{D}\left(0\right)=1/S_{s}$, in the analysis of single-cell transplantation assay data. Focusing on the differentiation of HSCs into progenitor cells, the capacity to produce ST-HSCs from LT-HSCs, $p_{LS}$, increased with age (Figure 3A), but the capacity to produce progenitor cells of each blood cell lineage from ST-HSCs, $p_{SN},p_{SN},p_{SE}\text{,}$ and $p_{SP}$, decreased (Figure 3B). In particular, the capacity to produce the myeloid lineage (neutrophils/monocytes, erythrocytes, and platelets) was greatly reduced ($p_{SN},p_{SE}\text{,}$ and $p_{SP}$ were 0.260, 0.512, and 0.431 times lower in aged HSCs, Table S1 and Figure 3B). Myeloid bypass tended to increase platelet production capacity, $p_{LP}$, which was 1.44 times higher in aged HSCs (Table S1 and Figure 3A). On the other hand, neutrophil/monocyte and erythrocyte production, $p_{\mathrm{LN}}$ and $p_{LE}$, tended to be low even in young LT-HSCs and decreased further with age (Figure 3A). The values of production capacity from HSCs to progenitors, $p_{\mathrm{LN}}$, $p_{LE}$, $p_{LP}$, $p_{SB},p_{SP},p_{SE}\text{,}$ and $p_{SN}$, represent the ratio of weekly production to the size of the progenitor population. The bypass pathways to produce neutrophils/monocytes and erythrocytes from LT-HSCs, $p_{LE}$, $p_{LP}$, were suggested to be largely inactive because their values were less than $10^{-6}$ per week even for young HSCs. The estimated HSC capacity and their age-related changes are summarized in Figure 3C.

Although changes in the self-renewal capacity of HSCs with aging and the degree of differentiation as a transition between populations have been quantified, we need to examine changes in the number of cells produced as a result of differentiation to assess the differentiation potential of HSCs. To quantify how age-related changes in LT-HSCs affect blood cell production, we compared the amount of each cell lineage produced from donor cells at each time point calculated with estimated individual parameters. Comparing the influx of mature cells from young and aged HSCs, we found that the production of B cells, $d_{B}E_{B2,D}\left(24\right)$, significantly decreased with age (the mean of log_10_ values were 1.55 and 0.730 for young and aged HSCs, respectively, the *p*-value was0.00119), whereas the production of myeloid cells, $\alpha_{i}d_{i}Y_{i,D}$ (i=N,E,P), remained the same (the mean of log_10_ values were 1.80, 5.42 and 4.60 for young and 1.37, 5.25 and 4.36 for aged HSCs, Figure 3D). The ratio of the production of the three myeloid cell lineages to that of B cells as representative of lymphocytes, $\alpha_{i}d_{i}Y_{i,D}\left(t\right)/d_{B}E_{B2,D}\left(t\right)$ for i=N,E,P, tended to increase with age in all lineages throughout transplantation (Figure 3E). These ratios at week 24, $\alpha_{i}d_{i}Y_{i,D}\left(24\right)/d_{B}E_{B2,D}\left(24\right)$ for i=N,E,P, were significantly increased in all lineages (the mean of log_10_ values were 0.253, 3.87 and 3.05 for young and 0.637, 4.52 and 3.63 for aged HSCs, Figure 3F), which is consistent with the myeloid skewing of HSCs with aging reported in another study.^19^ The above results suggest that, at least under transplantation conditions, a myeloid shift in HSCs with aging was confirmed as the absolute number of mature blood cells. This skewing toward myeloid differentiation was caused by decreased lymphoid production rather than by increased myeloid production.

The HSC transplantation assay has been used to evaluate the self-renewal and differentiation potential of HSCs. The magnitude of chimerism is sometimes regarded as the strength of the self-renewal or differentiation of HSCs; however, given the mechanism of the transplantation system, it may also reflect the differentiation potential of the blood cell lineage being observed and the characteristics of competing cells. The estimated self-renewal rate of LT-HSCs, which is expressed as an intrinsic growth rate in our model, $r_{L}$, was slightly positively correlated with chimerism at 24 weeks in B cells only (Figure S5A). However, there was much variability, and it seems unlikely that a linear relationship explains this relationship (Figures S5B and S5C). The number of LT-HSCs in the competitor cells, which encompasses not only the number relative to transplanted single cells but also relative capacity, showed a slight negative correlation except for B cells (Figure S5C). However, there was a large variation in this metric as well (the second column from the right in Figure S5A). The strongest correlation was found between the intensity of B cell production from ST-HSC, $p_{SB}$, and B cell chimerism (Figure S5C), which may still explain at most 49.0% of B cell chimerism (Figure S5B). The capacity of individual HSCs must be evaluated in combination with their ability to produce blood cells of multiple lineages and in the process of differentiation to peripheral blood. In subsequent sections, we quantitatively evaluated the long-term reconstitution capacity of HSCs as their functional hematopoietic capacity, focusing on time-dependent changes in production at each stage of HSC differentiation, or influx, and in chimerism as experimental output in transplantation assays.

### Changes in the degree of dependence of platelets on myeloid bypass represent differences in the time course of mature cell production

**Figure 4:**
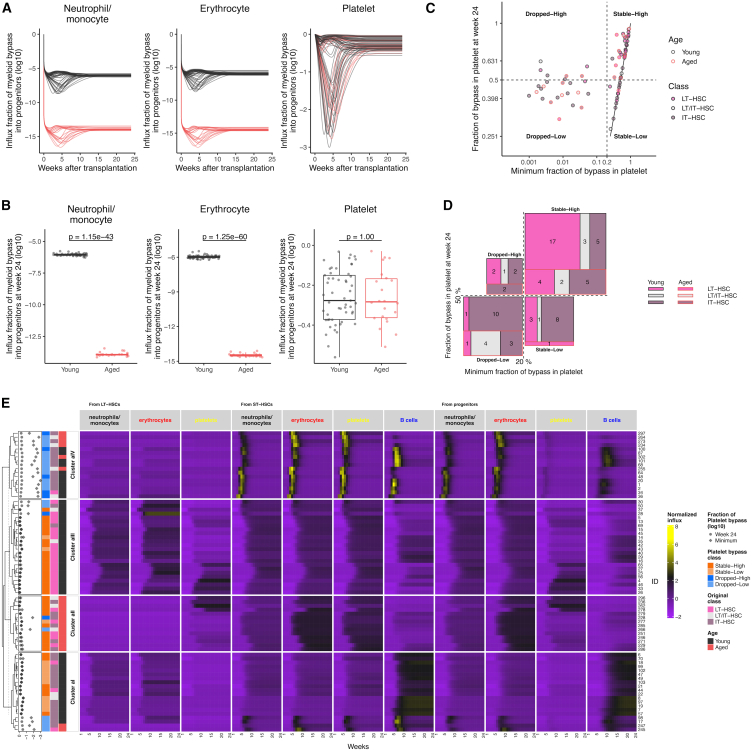
Dependence on the bypass pathway in myeloid cell production and HSC skewing in differentiation (A and B) Dependency on the bypass pathway in each lineage of myeloid cells at each time after transplantation (i.e., $p_{Li,D,k}L_{D,k}\left(t\right)/\left(p_{Li,D,k}L_{D,k}\left(t\right)+p_{Si,D,k}S_{D,k}\left(t\right)\right)$ (i=N,E,P and $k=n_{L1},...,n_{L75}$), (A)) and at 24 weeks after transplantation (i.e., $p_{Li,D,k}L_{D,k}\left(24\right)/\left(p_{Li,D,k}L_{D,k}\left(24\right)+p_{Si,D,k}S_{D,k}\left(24\right)\right)$ (i=N,E,P and $k=n_{L1},...,n_{L75}$), (B)). (C) Correlation of the minimum dependency of platelet production on the bypass pathway in each transplantation assay and dependency at 24 weeks after transplantation. Solid vertical and horizontal lines show the dependency at 50% and 20%, respectively. (D) Relationship between classes of HSCs defined by platelet bypass and aging, and classes defined by reconstitution capacity. The number of HSCs of the type indicated by the color corresponds to the size of the rectangle, which corresponds to (C) and which are summarized in Table 2. (E) Hierarchical clustering of the production of each cell population. The values at 24 weeks after transplantation (red) and the minimum (blue) for the fraction of platelet bypass in the platelet progenitor production are shown next to the dendrogram. The left side of the production heatmap shows the class defined by platelet bypass, the class defined by the duration of reconstitution after transplantation, and of the transplanted HSCs. P-values in (B) were calculated by the Student’s t test for the mean of young and aged HSCs with Bonferroni correction and significance level as 0.05.

The dependence on the bypass pathway in differentiation from LT-HSCs into myeloid lineages was defined as the fraction of influx into progenitor cells that was not via ST-HSCs in the overall influx in single-LT-HSC transplantations (i.e., $p_{Li,D}L_{D}\left(t\right)/\left(p_{Li,D}L_{D}\left(t\right)+p_{Si,D}S_{D}\left(t\right)\right)$, Figure 4A). Since the calculated blood cell production from donor cells was found to be roughly steady-state at 24 weeks after transplantation (Figure S6), we compared the fraction at week 24 as a property of HSCs in the post-reconstitution of hematopoiesis. The degree of dependence on myeloid bypass at week 24 was higher for platelets than for neutrophils/monocytes and erythrocytes (Figures 4A and 4B). The dependency on the bypass pathway at week 24 for neutrophils/monocytes and erythrocytes production was suggested to decrease with age (the *p*-values were $1.15\times 10^{-43}$ and $1.25\times 10^{-60}$, respectively), and were quite low, less than $10^{-5}$, even in young HSCs (Figures 4A and 4B). The platelet dependency on the bypass pathway at week 24 had no significant difference with age. In the process of blood cell reproduction from a single cell, the degree of dependence on platelet bypass changed with time after transplantation (Figure 4A). Particularly notable was the decrease in the degree of dependence on platelet bypass up to 10 weeks after transplantation in some HSCs, regardless of the degree of dependence at week 24 or the age of the transplanted HSCs (Figures 4A and 4C). Based on the difference in dependency on platelet bypass, HSCs can be divided into two categories: HSCs with a degree of dependence on the bypass pathway of less than 20% at any time point were defined as “dropped,” and all other HSCs were defined as “stable.” To also take into account the dependency at week 24 compared above, we defined HSCs with dependency lower than 50% at 24 weeks after transplantation as “low,” and the rest as “high” (Figure 4C). The majority of LT-HSCs were stable-high (21 of 29 LT-HSCs), suggesting a correlation between long-term reconstitution capacity and dependence on platelet bypass (Figure 4D and Table 2). HSCs defined as dropped were IT-HSCs (22 of 26 HSCs defined as dropped, including LT/IT-HSCs).

**Table 2:** Platelet bypass dependency in single-cell transplantation

Age	Young (*N* = 53)			Aged (*N* = 22)		
Class	LT-HSC	LT/IT-HSC	IT-HSC	LT-HSC	LT/IT-HSC	IT-HSC
Stable-High	17	3	5	4	2	5
Stable-Low	3	1	8	1	0	0
Dropped-High	2	1	2	0	0	2
Dropped-Low	1	0	10	1	4	3

In addition to long-term reconstructive capacity, the skewing of hematopoiesis, i.e., which strains are relatively produced, is important in assessing the HSC differentiation. Hierarchical clustering was performed to unbiasedly classify LT-, LT/IT-, and IT-HSCs using relative blood cell production within individuals. To consider the entire HSC to mature cell differentiation, we calculated the influx of each cell population in our mathematical model using the estimated parameters (Figure S7). The influx at each time was standardized within each cell population, normalized within each individual, and used for clustering (Figure 4E **and** STAR Methods). Clusters classified by relative blood cell production clearly distinguished hematopoietic reconstitution capacity. LT-HSCs, where most of them were classified as cluster aIII, produced a large number of myeloid lineages in the late period. Most aged HSCs are classified as cluster aII and have relatively high erythrocyte production. Furthermore, within the IT-HSCs, a population with predominant B-cell production in the late period after transplantation (Cluster aI) and a population with peak blood cell production in the early period after transplantation (Cluster aIV) were identified. Based on these clusters, we compared the relationship between the skewing of blood cell production and the class defined by dependence on platelet bypass. Interestingly, the class of platelet bypass was clearly mapped to these clusters. Cluster aI is stable-low, Cluster aII and Cluster aIII are stable-high, and Cluster aIV is dropped class. These comparisons suggested that differences in platelet production pathways may be a surrogate indicator of overall skewing and reconstructive capacity of HSC blood cell production.

### Platelet bypass is correlated with platelet-biased potential compared to erythrocyte

To date, pHSCs have been classified by combining reconstitution duration data in primary and secondary transplants (LT, IT, or ST) and the extent of differentiation skewing to neutrophils/monocytes, B cells, and T cells at week 20–24 in the primary transplants (myeloid-biased, lymphoid-biased, or balanced). We wondered whether the dependence on platelet bypass that we found to be associated with reconstitution capacity and aging could be defined from the value of the data in the transplantation assay. We first aimed to characterize these large PB chimerism datasets by using a clustering approach.

**Figure 5:**
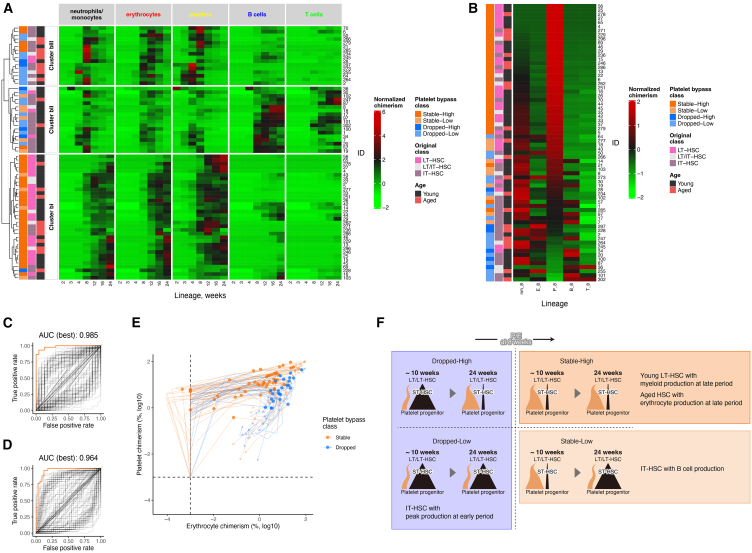
HSCs in the stable class of platelet bypass have platelet-biased potential (A) Hierarchical clustering of chimerism of each cell lineage observed in the transplantation assay. Each transplanted individual was divided into three groups by the k-means method. (B) Normalized chimerism at 8 weeks after transplantation with HSCs classified as LT-HSC or IT-HSC. The individuals are sorted in decreasing order using the normalized chimerism value of the platelet. The class defined by platelet bypass, the class defined by the duration of reconstitution after transplantation, and age for transplanted HSCs are shown next to the heatmap in (A) and (B). (C and D) ROC curves obtained for the classification of two classes, stable and dropped (C), and stable-high and others (D), defined by platelet bypass dependency with ratio of chimerism as an explanatory variable. Each line was obtained by changing the threshold for the combination of lineages of chimerism data and observation time used for classification and group assignment. The purple line shows the ROC curve obtained by using the ratio of erythrocyte to platelet chimerism at 8 weeks after transplantation, when the area under the curve (AUC) was highest. (E) Time change of erythrocyte and platelet chimerism values and class of platelet bypass. Erythrocyte and platelet chimerism in transplantation assays classified into stable and dropped classes based on platelet bypass dependency is shown by orange and black lines. The direction of the arrow indicates the passage of time after transplantation. The dots show chimerism values for platelets and erythrocytes at 8 weeks after transplantation. (F) Illustration of the transition in platelet production and the distinction between each class defined by platelet bypass. The orange and black bands indicate production from the bypass pathway and the pathway via ST-HSCs, respectively.

Hierarchical clustering of PB chimerism data from 68 single-cell transplantations, excluding 8 individuals containing missing observations, revealed three distinct clusters, Cluster bI, bII, and bIII (Figure 5A and STAR Methods). The three clusters were divided into two main groups according to the time of myeloid predominance. In Cluster bI, the chimerism of myeloid cells was predominant in the latter 12 to 24 weeks after transplantation. In contrast, Clusters bII and bIII had a predominance of myeloid cells in the first half of the transplantation, from 4 to 12 weeks. Clusters bII and bIII were distinguished by the predominance of lymphocytes at 12–24 weeks. This clustering could capture a particular lineage skewing in chimerism at a certain time point after transplantation. Interestingly, our analysis separated LT- and IT-HSCs into two major populations, Cluster bI and Cluster bII/bIII, respectively. Cluster bI mainly consisted of HSCs defined as stable-high for platelet bypass, whereas the remaining stable-highs belonged to Clusters bII and bIII and were all IT-HSC. Myeloid-biased output based on chimerism in Cluster bI related to long-term reconstituting capacity with stable platelet production from the bypass pathway. Cluster bII consisted of dropped and stable-low, resulting in lymphoid-biased output on chimerism. This suggests that the class defined by the degree of dependency on platelet bypass reflects not only the reconstitution capacity of HSCs, but also the skewing of chimerism as time changes of relative values between blood cell lineages.

We investigated the relationship between the platelet bypass and chimerism data at each time point after transplantation based on platelet dominance using relative values of chimerism at 2, 3, 4, 8, 12, 16, and 24 weeks. To assess skewing in chimerism for the five observed lineages, we normalized the chimerism values to follow a distribution with a mean of 0 and variance of 1 within each individual. The values were then sorted in descending order by normalized chimerism of platelet values, indicating platelet predominance, and examined in relation to other blood cell lineages and classes defined by platelet bypass (Figures 5B and S8). This analysis revealed that relative values of platelet chimerism at 4 to 24 weeks correlated highly with the stable-high class for platelet bypass (Figures 5B and S8), especially suggesting that the values at 8 weeks separate the stable and dropped classes regardless of whether the class was high or low (Figures 5B). In addition, the stable and dropped classes were observed to correlate inversely with relative values of chimerism in lineages other than platelets at 8 weeks after transplantation (Figure 5B). These findings demonstrate the possibility of classifying HSCs with classes of platelet bypass that encompass the reconstitution capacity and the bias in the blood cell production of HSCs by examining skewing in chimerism at a given time point in transplantation assays.

The analysis of the pHSC population so far suggests that platelet bypass is important for functional HSCs. Therefore, we searched for features that could classify the stable and dropped classes of platelet bypass that could roughly distinguish functional LT-HSCs from IT-HSCs. In particular, we comprehensively investigated whether the ratio of chimerism could separate the stable from the dropped class, since the skewing in HSC blood cell production and chimerism data was suggested to be relevant to the classes. For all combinations of the five cell lineages and observation times, we evaluated the accuracy and specificity for the stable and dropped classifications based on the chimerism ratio of two lineages with varying thresholds. Based on the area under the curve (AUC) of the receiver operating characteristic (ROC) curve, we found that the ratio of platelet to erythrocyte chimerism at 8 weeks after transplantation best separated the stable from the dropped class (AUC was 0.979, shown in purple line in Figure 5C). The classification of stable-high, to which most LT-HSCs belonged, and other classes was equally possible (AUC was 0.95, shown in purple line in Figure 5D). The time variation of the combination of erythrocyte and platelet chimerism values differed between the stable and dropped classes, with a clear difference at week 8 (circles in Figure 5E), whereas platelet values were relatively higher in the stable class and erythrocyte values were higher in the dropped class. Taken together, these data indicate that platelet bypass is strongly correlated with skewing toward platelet compared with erythrocyte chimerism. The data also imply that the dependency on platelet bypass defined by mathematical modeling can be estimated by the chimerism ratio of platelet versus erythrocyte at 8 weeks before the long-term observation of chimerism (Figure 5F).

## Discussion

Here, we have elucidated how and when transplanted HSCs use the platelet bypass pathway through mathematical analysis of PB chimerism datasets. We performed this analysis without using the paired-daughter cell assay and without analyzing cell division in the bone marrow (BM). Almost all LT-HSCs showed stable dependency on platelet bypass throughout the analysis (up to 24 weeks). On the other hand, we found that the dependency on neutrophil/monocyte bypass and erythrocyte bypass decreased with aging and that the absolute amounts of production via the bypass pathway were low. When we compared the classes of dependency on platelet bypass, we could identify HSCs with the stable functioning of platelet bypass (AUC of ROC curve was 0.985) with the use of a simple formula calculating the ratio of platelet to erythrocyte chimerism at 8 weeks. Stable platelet production via the bypass pathway may be a requirement for long-term blood cell renewal as LT-HSCs (especially young LT-HSCs), and this new property can be determined by use of transplantation assays of relatively short duration. By investigating cell populations in the BM after HSC transplantation, we can clarify the differentiation kinetics of HSCs with greater certainty.

Whether the relationship we have found between the use of the platelet bypass pathway and long-term hematopoietic reconstitution is a phenomenon under the special conditions of transplantation needs to be investigated. The presence of stem-like megakaryocyte-committed progenitors (SL-MkPs) activated by inflammation has been suggested,^20^ while LT-HSCs in native hematopoiesis, or unperturbed hematopoiesis, have been shown to be part of the source of megakaryocyte-restricted progenitors.^21^ The age-specific emergence of a pathway for the direct production of platelets and the presence of MkPs with different activities produced by two different pathways have been demonstrated.^22^^,^^23^ Although our study did not explicitly consider differences in proliferation capacity or lifespan of platelet lineages by pathway of production, a mathematical model that considers differences in the characteristics of the produced blood cell population may be useful for elucidating the causes of related diseases and evaluating therapeutic interventions.

The percentage of cells derived from donor cells in PB in transplantation has been used by itself to assess reconstitution capacity and differentiation skewing, but we found that those values did not always reflect the estimated HSC self-renewal and differentiation potential. Given that our model also includes the effects of competitive proliferation with competitor cells, the mathematical modeling analysis gives a more precise assessment of the differentiation potential of HSCs. In particular, we showed a relative predominance of myeloid production by aged HSCs, with a decrease in B cell production. Several transplantation assays, transcriptome analysis, and tracking by fluorescent protein and genetic barcoding have shown a bias of the HSCs themselves toward myeloid differentiation and myeloid-dominant blood cell production in mice and humans.^12^^,^^13^^,^^19^^,^^24^^,^^25^^,^^26^^,^^27^^,^^28^^,^^29^ Our estimated and calculated results are also consistent with the results of these previous studies. It is a finding that, at least in the transplantation setting, the absolute amount of myeloid production does not change.

Previously, we elucidated a differentiation pathway of HSCs using a paired-daughter cell assay, where two daughter cells after one division were transferred into separate mice to compare *in vivo* differentiation potential.^4^ This is the only way to compare the differentiation potential of the two daughter cells *in vivo*; however, a single cell needs to divide *in vitro*. Our approach, combining *in vivo* data with mathematical modeling, has successfully quantified how this pathway could work in a transplantation setting. Our mathematical model, which captures the kinetics of blood cell production, predicted time-dependent changes in dependence on the bypass pathway and revealed changes in HSC reconstitution capacity with age.

So far the mechanisms underlying myeloid (especially platelet) bypass remain to be elucidated. Direct comparison between daughter cells after one division of HSCs may help to identify these mechanisms. However, we cannot estimate whether the HSC divides via myeloid bypass or not. In other words, we cannot analyze which HSCs will pass through the myeloid bypass. One approach would be to analyze the gene expression of many daughter cells when HSCs divide, but their gene expression cannot be mapped to their reconstitution capacity. Exhaustive screening is also difficult, but the model derived in this study should make this possible. Our simple calculation can identify platelet bypass at 8 weeks after transplantation. If we focus on the HSCs that differentiate to give rise to platelets via platelet bypass, we can detect candidate molecules to promote or inhibit platelet bypass. Using this method, we will be able to obtain candidate genes that will affect platelet bypass dependency after CRISPR screening.

In conclusion, the approach developed here aids in the quantitative understanding of HSC differentiation and redefines how HSC competence is assessed. The dependence on platelet bypass and its time- and age-related changes highlighted by our findings may contribute to a better understanding of the stemness and differentiation of HSCs.

### Limitations of the study

The differentiation hierarchy of the model from LT-HSCs to progenitors is debatable. It is not clear whether ST-HSCs always differentiate from LT-HSCs. In this model, the production of ST-HSCs from LT-HSCs in competitor cells results in single ST-HSCs that are eliminated, representing their short blood cell production. Blood cell production via multipotent progenitors, common myeloid progenitors, and common lymphoid progenitors can all be interpreted as rounding to production from ST-HSCs to progenitors. The results for platelet bypass were obtained by focusing only on the bypass pathway from LT-HSCs. The existence of bypass pathways from more differentiated stages, such as ST-HSCs in this case, in the sense that they do not go through a common progenitor cell, is not ruled out and these pathways may play an important role.

In transplantation assays, the percentage of donor cells in the PB cannot be measured if the cells are not engrafted. Given that we observed blood cell production from a very small number of HSCs, i.e., single cells; it is possible that there is a stochastic failure to engraft. Therefore, the differentiation kinetics of HSCs estimated in this study may be the result of a survival bias of those that happen to be reconstitutive in single-cell transplantation. Even in light of these limitations, our results captured the dynamics of hematopoiesis during HSC reconstitution and provided insights, especially for platelet bypass.

## Resource availability

### Lead contact

Requests for further information and resources should be directed to and will be fulfilled by the lead contact, Ryo Yamamoto (yamamoto.ryo.2c@kyoto-u.ac.jp).

### Materials availability

This study did not generate new unique reagents.

### Data and code availability

• The chimerism and CBC data have been deposited at Zenodo and are publicly available as of the date of publication. Accession numbers are listed in the key resources table.
• Parameter estimation codes have been deposited at Zenodo and are publicly available as of the date of publication. Accession numbers are listed in the key resources table.
• Any additional information required to reanalyze the data reported in this article is available from the lead contact upon request.

## Acknowledgments

The authors thank Jennifer Holmes for providing language editing support. This study was supported in part by Scientific Research (KAKENHI) 22K15073 (to S.Iwanami); 23H03497 (to S. Iwami); Grant-in-Aid for Transformative Research Areas 22H05215 (to S. Iwami); Grant-in-Aid for Challenging Research (Exploratory) 22K19829 (to S. Iwami); AMED CREST 19gm1310002 (to S. Iwami); AMED Research Program on Emerging and Re-emerging Infectious Diseases 22fk0108509 (to S. Iwami), 23fk0108684 (to S. Iwami), 23fk0108685 (to S. Iwami); AMED Research Program on HIV/AIDS 22fk0410052 (to S. Iwami); AMED Program for Basic and Clinical Research on Hepatitis 22fk0210094 (to S. Iwami); AMED Program on the Innovative Development and the Application of New Drugs for Hepatitis B 22fk0310504h0501 (to S. Iwami); JST PRESTO Grant Number JPMJPR21R3 (to S. Iwanami); MIRAI JPMJMI22G1 (to S. Iwami); Moonshot R&D JPMJMS2021 (to S. Iwami) and JPMJMS2025 (to S. Iwami); Shin-Nihon of Advanced Medical Research (to S. Iwami); 10.13039/501100004298SECOM Science and Technology Foundation (to S. Iwami); The Japan Prize Foundation (to S. Iwami); Suntory Rising Stars Encouragement Program in Life Sciences (SunRiSE) (R.Y.).

## Author contributions

Conceptualization, S. Iwami and R.Y.; methodology, S. Iwanami, T.S., H.H., L.C., X.L. and S. Iwami; software, S. Iwanami, T.S. and L.C.; validation, S. Iwanami, H.H. and S. Iwami; formal analysis, S. Iwanami and T.S.; resources, H.N. and R.Y.; data curation, J.O. and R.Y.; investigation, S. Iwanami, T.S., H.H., S. Iwami and R.Y.; writing – original draft, S. Iwanami, K.I., S. Iwami and R.Y.; writing – review and editing, S. Iwami and R.Y.; visualization, S. Iwanami and T.S.; supervision, S. Iwami and R.Y.; project administration, S. Iwami and R.Y; funding acquisition, S. Iwanami, H.H., S. Iwami and R.Y.

## Declaration of interests

H. N. is a co-founder and shareholder in Megakaryon, Century Therapeutics and Celaid Therapeutics. The remaining authors declare no competing interests.

## STAR★Methods

### Key resources table

**Table:** 

REAGENT or RESOURCE	SOURCE	IDENTIFIER
**Deposited data**		

Chimerism and CBC data	Zenodo	https://doi.org/10.5281/zenodo.14722503

**Software and algorithms**		

MonolixSuite2024R1	Lixoft SAS	https://doi.org/10.5281/zenodo.11401936
R 4.4.1	R Core Team	https://www.R-project.org/
Parameter estimation code	Zenodo	https://doi.org/10.5281/zenodo.14722485

### Method details

#### Dataset of chimerism and CBC in single-cell transplantation

The longitudinal chimerism data from single-cell transplantation were previously published.^4^^,^^5^^,^^15^ Chimerism was measured as the fraction of KuO^+^ cells in each cell lineage—neutrophil/monocyte, erythrocyte, platelet, B cell, and T cell—after transplantation of young and aged KuO^+^ phenotypically defined HSCs (CD150^+^CD41^-^CD34^-^KSL, CD150^+^CD41^+^CD34^-^KSL or CD150^-^CD34^-^KSL, pHSCs) into lethally irradiated mice. A total of $2\times 10^{5}$ BM cells were transplanted along with single HSCs in these assays. Single transplanted cells in which chimerism was above the threshold (0.1% or 0.005%) in at least one measurement after primary transplantation in all five blood cell lines was classified as an HSC. Of the transplantation assays in which chimerism was above threshold in all five lines at the end of the primary transplantation, those in which chimerism was above threshold in all five lines throughout the secondary transplant were defined as long-term (LT-) HSCs, and others in which chimerism was below threshold in at least one blood cell lineage at the secondary transplantation were intermediate-term (IT-) HSCs. If the primary transplantation met the criteria for LT- or IT-HSCs and no second transplantation was performed, single cells were treated as LT/IT-HSCs, since LT-HSC and IT-HSC were indistinguishable. HSCs with chimerism below threshold in at least one blood cell lineage at the end of the primary transplantation were defined as short-term (ST-) HSCs. Single cells obtained from 8- to 12-week-old and 20- to 24-month-old and classified as HSCs were designated as young HSCs and aged HSCs, respectively. For the estimation of our model parameters, we used the data from primary transplantations in which transplanted single cells were classified as LT-HSCs, IT-HSCs, LT/IT-HSCs, and ST-HSCs (Table 1; Figures 1B and S1). Since it is not possible to distinguish between LT- and IT-HSC based on primary transplantation data alone, we assumed that LT-, IT- and LT/IT-HSC belong to the LT-HSC population in our mathematical model. The data with measurements less than three points in at least one blood cell lineage were excluded. The chimerism data of T cells were excluded for parameter estimation to avoid the complexity of the mathematical model to describe differentiation into mature T cells, which is expected to be slower than in other lineages, and the estimation of a large number of parameters. CBCs measured in a subset of experiments in which the chimerism data met the above criteria were also used to estimate parameters for the mathematical model (Table 1 and Figure S2).

#### Recipient chimerism in BM cell transplantation

To consider the effect of the percentage of the recipient's original cells on erythrocyte and platelet chimerism in single-cell transplantation assays, transplantations of KuO-labeled BM cells into lethally irradiated mice were conducted as additional experiments (Table 1). As in the single-cell transplant experiment, the fraction of donor and recipient cells in erythrocytes and platelets in the PB after transplantation was measured.^15^ The percentage of recipient-derived cells was used for fitting the mathematical model (Figure S3).

#### HSC differentiation model

To analyze the dynamics of chimerism in the single-cell transplantation assays, we developed a differentiation model of HSCs. In the base model, self-renewing LT-HSCs differentiate into ST-HSCs, which then differentiate into mature cells through progenitors. Using ordinary differential equations, the mathematical model is described as follows:

(Equation 1) $$\frac{d\bar{L}\left(t\right)}{dt}={\bar{r}}_{L}\left(1-\frac{\bar{L}\left(t\right)}{{\bar{K}}_{L}}\right)\bar{L}\left(t\right)$$

(Equation 2) $$\frac{d\bar{S}\left(t\right)}{dt}={\bar{r}}_{S}\left(1-\frac{\bar{S}\left(t\right)}{{\bar{K}}_{S}}\right)\bar{S}\left(t\right)+{\bar{p}}_{LS}\bar{L}\left(t\right)$$

(Equation 3) $$\frac{d{\bar{S}}_{i}\left(t\right)}{dt}={\bar{p}}_{Li}\bar{L}\left(t\right)+{\bar{p}}_{Si}\bar{S}\left(t\right)-{\bar{d}}_{i}{\bar{Y}}_{i}\left(t\right)\;\left(i=N,E,P,B\right)$$

(Equation 4) $$\frac{d{\bar{Z}}_{i}\left(t\right)}{dt}={\bar{\alpha }}_{i}{\bar{d}}_{i}{\bar{Y}}_{i}\left(t\right)-{\bar{\delta }}_{i}{\bar{Z}}_{i}\left(t\right)\;\left(i=N,E,P,B\right)$$

The variables $\bar{L}\left(t\right)$ and $\bar{S}\left(t\right)$ represent the number of LT-HSCs and ST-HSCs at time t, and the numbers of progenitors and mature cells at time t in neutrophils/monocytes (i=N), erythrocytes (i=E), platelets (i=P), and B cells (i=B) are represented by ${\bar{Y}}_{i}\left(t\right)$ and ${\bar{Z}}_{i}\left(t\right)$, respectively. The ability of LT-HSCs and ST-HSCs to proliferate by self-replication is described as logistic proliferation using the intrinsic growth rate, ${\bar{r}}_{L}$ and ${\bar{r}}_{S}$, and the carrying capacity ${\bar{K}}_{L}$ and ${\bar{K}}_{S}$, respectively. ST-HSCs are produced from LT-HSCs at rate ${\bar{p}}_{LS}$, and progenitor cells are produced from ST-HSCs and ST-HSCs at rates ${\bar{p}}_{Li}$ and ${\bar{p}}_{Si}$ (i=N,E,P,B), respectively. Direct production of progenitor cells from LT-HSCs, ${\bar{p}}_{Li}\bar{L}\left(t\right)$, represents a bypass pathway with lineage-restricted differentiation. Here ${\bar{p}}_{LB}=0$ because we consider only the bypass pathway for differentiation into myeloid lineages. Progenitors differentiate at rate ${\bar{d}}_{i}$ (i=N,E,P,B) and mature cells are produced with expansion rate ${\bar{\alpha }}_{i}$ (i=N,E,P,B). Mature cells die at rate ${\bar{\delta }}_{i}$ (i=N,E,P,B).

In normal blood cell production, all populations are considered to be in steady-state, which is described as $\frac{d\bar{L}\left(t\right)}{dt}=0$, $\frac{d\bar{S}\left(t\right)}{dt}=0$, $\frac{d{\bar{Y}}_{i}\left(t\right)}{dt}=0$, $\frac{d{\bar{Z}}_{i}\left(t\right)}{dt}=0$ and $\bar{L}\left(t\right)=L^{*}\text{,}$ $\bar{S}\left(t\right)=S^{*}$, ${\bar{Y}}_{i}\left(t\right)={Y_{i}}^{*}$, ${\bar{Z}}_{i}\left(t\right)={Z_{i}}^{*}$, where $L^{*}={\bar{K}}_{L}$. Using these relationships, (Equation 1), (Equation 2), (Equation 3), (Equation 4) can be transformed as follows:

(Equation 5) $$\frac{dL\left(t\right)}{dt}=r_{L}\left(1-L\left(t\right)\right)L\left(t\right)$$

(Equation 6) $$\frac{dS\left(t\right)}{dt}=r_{S}\left(1-\frac{S\left(t\right)}{K_{S}}\right)S\left(t\right)+p_{LS}L\left(t\right)$$

(Equation 7) $$\frac{dY_{i}\left(t\right)}{dt}=p_{Li}L\left(t\right)+p_{Si}S\left(t\right)-d_{i}Y_{i}\left(t\right)\;\left(i=N,E,P,B\right)$$

(Equation 8) $$\frac{dZ_{i}\left(t\right)}{dt}=\alpha_{i}d_{i}Y_{i}\left(t\right)-\delta_{i}Z_{i}\left(t\right)\;\left(i=N,E,P,B\right)$$

Here, the variables are converted as $L\left(t\right)=\frac{\bar{L}\left(t\right)}{L^{*}}$, $S\left(t\right)=\frac{\bar{S}\left(t\right)}{S^{*}}$, $Y_{i}\left(t\right)=\frac{{\bar{Y}}_{i}\left(t\right)}{{Y_{i}}^{*}}$, $Z_{i}\left(t\right)={\bar{Z}}_{i}\left(t\right)$. Thus, the parameters are converted as $r_{L}={\bar{r}}_{L}$, $r_{S}={\bar{r}}_{S}$, $K_{S}=\frac{r_{S}}{p_{LS}+r_{S}}$, $p_{LS}=\frac{L^{*}}{S^{*}}{\bar{p}}_{LS}$, $p_{Li}=\frac{L^{*}}{{Y_{i}}^{*}}{\bar{p}}_{Li}$, $p_{Si}=\frac{S^{*}}{{Y_{i}}^{*}}{\bar{p}}_{Si}$, $d_{i}={\bar{d}}_{i}$, $\alpha_{i}=\frac{{Y_{i}}^{*}}{{Z_{i}}^{*}}{\bar{\alpha }}_{Li}$, $\delta_{i}={\bar{\delta }}_{i}$. The steady-state relationship allows us to treat some parameters as $d_{i}=p_{Li}+p_{Si}$ and $\alpha_{i}=\frac{\delta_{i}{Z_{i}}^{*}}{d_{i}}$.

#### Mathematical model for single-cell transplantation

In the single-cell transplantation assay, BM cells (competitors) are transplanted at the same time as single cells (donor cells). In addition, recipient cells are included in the measurement of erythrocytes and platelets. The competitive proliferation of donor- (j=D) and competitor- (j=C) and recipient- (j=R) derived cells in the single-cell transplantation assay can be expressed by extending (Equation 5), (Equation 6), (Equation 7), (Equation 8) as follows:

(Equation 9) $$\frac{dL_{j}\left(t\right)}{dt}=r_{L,j}\left(1-\sum_{m=\left\{D,C,R\right\}}L_{m}\left(t\right)\right)L_{j}\left(t\right)$$

(Equation 10) $$\frac{dS_{j}\left(t\right)}{dt}=r_{S,j}\left(1-\frac{\sum_{m=D,C,R}S_{m}\left(t\right)}{K_{S}}\right)S_{j}\left(t\right)+p_{LS,j}L_{j}\left(t\right)$$

(Equation 11) $$\frac{dY_{i,j}\left(t\right)}{dt}=p_{Li,j}L_{j}\left(t\right)+p_{Si,j}S_{j}\left(t\right)-d_{i}Y_{i,j}\left(t\right)$$

(Equation 12) $$\frac{dZ_{i,j}\left(t\right)}{dt}=\alpha_{i}d_{i}Y_{i,j}\left(t\right)-\delta_{i,j}Z_{i,j}\left(t\right)\;\left(i=N,E,P\right)$$

(Equation 13) $$\frac{dE_{B1,j}\left(t\right)}{dt}=\alpha_{B}d_{B}Y_{B,j}\left(t\right)-d_{B}E_{B1,j}\left(t\right)$$

(Equation 14) $$\frac{dE_{B2,j}\left(t\right)}{dt}={d_{B}E}_{B1,j}\left(t\right)-d_{B}E_{B2,j}\left(t\right)$$

(Equation 15) $$\frac{dZ_{B,j}\left(t\right)}{dt}=d_{B}E_{B2,j}\left(t\right)-\delta_{B,j}Z_{B,j}\left(t\right)$$

where t is the time after transplantation. The subscript j in each variable and parameter corresponds to the cell origin (donor cells, competitors, recipient cells). Here, we assume the existence of an eclipse phase in the differentiation of progenitor cells to mature cells to represent the slow production of B cells. This delay is explained by the commonly used linear chain trick. Since the recipient mice have been lethally irradiated and are not capable of producing blood cells, the parameters other than the death rate of mature cells, $\delta_{i,j}$, related to the recipient-derived cells are zero. The differentiation of non-HSC populations, which are described by parameters $d_{i}$ and $\alpha_{i}$, is assumed to be the same for donor- and recipient-derived cells. Death of mature cells was assumed to be different only for recipient-derived cells as an effect of irradiation, $\delta_{i,D}=\delta_{i,C}$ and $\delta_{i,D}\neq \delta_{i,R}$. The effect of the competition between donor and competitor derived LT- and ST-HSCs on their proliferation was assumed to be the same because equivalent populations were considered in our mathematical model, (Equation 9), (Equation 10). The initial value of competitor derived LT- and ST-HSCs, $L^{*}$ and $S^{*}$ may include the strength of the competition as a ratio of the number of cells.

Chimerism and CBC were measured in the single-cell transplantation assay. In the measurements, donor cells are identified by KuO, a propensity protein. In neutrophils/monocytes and B cells, competitors and recipient cells are distinguished by the expression of Ly5.1 and Ly5.2, which are cell surface markers of lymphocytes, but not in erythrocytes and platelets. Thus, the chimerism, $F_{j}\left(t\right)$, and CBC, $T_{j}\left(t\right)$, for each lineage obtained in the experiment are calculated using (Equation 9), (Equation 10), (Equation 11), (Equation 12), (Equation 13), (Equation 14), (Equation 15) as follows:

(Equation 16) $$F_{i}\left(t\right)=100\times \frac{Z_{i,D}\left(t\right)}{Z_{i,D}\left(t\right)+Z_{i,C}\left(t\right)}\;\left(i=N,B\right)$$

(Equation 17) $$F_{i}\left(t\right)=100\times \frac{Z_{i,D}\left(t\right)}{Z_{i,D}\left(t\right)+Z_{i,C}\left(t\right)+Z_{i,R}\left(t\right)}\;\left(i=E,P\right)$$

(Equation 18) $$T_{i}\left(t\right)=Z_{i,D}\left(t\right)+Z_{i,C}\left(t\right)\;\left(i=N,B\right)$$

(Equation 19) $$T_{i}\left(t\right)=Z_{i,D}\left(t\right)+Z_{i,C}\left(t\right)+Z_{i,R}\left(t\right)\;\left(i=E,P\right)$$

In the single-cell transplantation assay, single cells in pHSCs are transplanted. The initial conditions of donor cells are $L_{D}\left(0\right)=1/L^{*}$, $S_{D}\left(0\right)=0$, $Y_{i,D}\left(0\right)=0$, $E_{B1,D}\left(0\right)=0$, $E_{B2,D}\left(0\right)=0$, $Z_{i,D}\left(0\right)=0$ or $L_{D}\left(0\right)=0$, $S_{D}\left(0\right)=1/S^{*}$, $Y_{i,D}\left(0\right)=0$, $E_{B1,D}\left(0\right)=0$, $E_{B2,D}\left(0\right)=0$, $Z_{i,D}\left(0\right)=0$ for the transplantations in which transplanted single cells are classified as LT-HSCs (including IT-HSCs) or ST-HSCs, respectively. Assuming that the $2\times 10^{5}$ BM cells transplanted as competitor cells contain a small amount of HSCs and progenitor cells that can be responsible for blood cell production, the initial condition was set to $L_{C}\left(0\right)=L_{C,0}$, $S_{C}\left(0\right)=S_{C,0}$, $Y_{i,C}\left(0\right)=1$, $E_{B1,C}\left(0\right)=0$, $E_{B2,C}\left(0\right)=0$, $Z_{i,C}\left(0\right)=0$. Since recipient mice were lethally irradiated, we adopted initial conditions, $L_{R}\left(0\right)=0$, $S_{R}\left(0\right)=0$, $Y_{i,R}\left(0\right)=0$, $E_{B1,R}\left(0\right)=0$, $E_{B2,R}\left(0\right)=0$, $Z_{i,R}\left(0\right)={Z_{i}}^{*}$, in which HSCs and progenitor cells were absent and assumed that they were unable to produce blood cells. The numbers of mature cells derived from recipient cells, ${Z_{i}}^{*}$, were estimated as averages of the values of CBC at 20 and 24 weeks after transplantation (Figure S9).

#### Mathematical model for BM cell transplantation

To quantify the kinetics of recipient-derived cells of erythrocytes and platelets, an experiment was performed in which BM cells labeled with KuO were transplanted. In this experiment, there are cells derived from BM cells, corresponding to the competitors of the single-cell transplantation assay, and recipient-derived cells. Chimerism was measured as the percentage of recipient-derived erythrocytes and platelets in PB, which was described using (Equation 9), (Equation 10), (Equation 11), (Equation 12), (Equation 13), (Equation 14), (Equation 15) as follows:

(Equation 20) $${\hat{F}}_{i}\left(t\right)=100\times \frac{Z_{i,R}\left(t\right)}{Z_{i,C}\left(t\right)+Z_{i,R}\left(t\right)}\;\left(i=E,P\right)$$

Assuming that the composition of the BM cells to be transplanted is similar to that of the competitors in the single-cell transplantation assay, the initial values for (Equation 9), (Equation 10), (Equation 11), (Equation 12), (Equation 13), (Equation 14), (Equation 15) are $L_{D}\left(0\right)=0$, $S_{D}\left(0\right)=0$, $Y_{i,D}\left(0\right)=0$, $E_{B1,D}\left(0\right)=0$, $E_{B2,D}\left(0\right)=0$, $Z_{i,D}\left(0\right)=0$, $L_{C}\left(0\right)=L_{C,0}$, $S_{C}\left(0\right)=S_{C,0}$, $Y_{i,C}\left(0\right)=1$, $E_{B1,C}\left(0\right)=0$, $E_{B2,C}\left(0\right)=0$, $Z_{i,C}\left(0\right)=0$, $L_{R}\left(0\right)=0$, $S_{R}\left(0\right)=0$, $Y_{i,R}\left(0\right)=0$, $E_{B1,R}\left(0\right)=0$, $E_{B2,R}\left(0\right)=0$, $Z_{i,R}\left(0\right)=Z_{i,R}\left(0\right)={Z_{i}}^{*}$ in BM cell transplantation.

#### Parameter estimation by nonlinear mixed-effect model

Using a nonlinear mixed-effects model, the parameters of the mathematical model were estimated from data obtained in single-cell and BM cell transplantation assays. Data fitting was implemented using MonolixSuite2024R1.^16^^,^^30^ A nonlinear mixed-effect model was used to fit the mathematical model for chimerism and CBC given by (Equation 18), (Equation 19), (Equation 20) to the dataset from the single-cell and BM cell transplantation assay. Assume that all parameters of differentiation kinetics of competitors are normal values of blood cell production (i.e., $r_{L,C}=r_{L}$, $r_{S,C}=r_{S}$, $p_{LS,C}=p_{LS}$, $p_{Li,C}=p_{Li}$, $p_{Si,C}=p_{Si}$). In addition, assuming that the transplanted single cells are heterogeneous in their proliferation and replication abilities, we further assumed that the values of their parameters vary around normal values. By introducing new scaling parameters with subscript d, the parameter for the differentiation kinetics of HSCs in donor cells is expressed as $r_{L,D}=r_{L,C}r_{L,d}$, $r_{S,D}=r_{S,C}r_{S,d}$, $p_{LS,D}=p_{LS,C}p_{LS,d}$, $p_{Li,D}=p_{Li,C}p_{Li,d}$, $p_{Si,D}=p_{Si,C}p_{Si,d}$. If the scaling parameters for individual k follow log-normal distributions and have differences between young and aged HSCs, then the parameters representing the ability of LT-HSCs, $\psi_{L,j}=\left\{r_{L,j},p_{LS,j},p_{Li,j}\right\}$ (j=D,C) and ST-HSCs, $\psi_{S,j}=\left\{r_{S,j},p_{Si,j}\right\}$ (j=D,C), for individual k follow the following formula:

(Equation 21) $$\log\left(\psi_{L,D,k}\right)=\log\left(\psi_{L,C}\right)+\beta_{age=aged,\psi_{L}}+\eta_{\psi_{L},k}\left(k=n_{L1},...,n_{L75}\right)$$

(Equation 22) $$\log\left(\psi_{S,D,k}\right)=\log\left(\psi_{S,C}\right)+\beta_{age=aged,\psi_{S}}+\eta_{\psi_{S},k}(k=n_{L1},...,n_{L75},n_{S1},...,n_{S39})$$

$\beta_{age=aged,\psi_{L}}$ and $\beta_{age=aged,\psi_{S}}$ are covariates for the ability of aged HSCs. $\eta_{\psi_{L},k}$ and $\eta_{\psi_{S},k}$ are random effects following normal distributions with mean 0 and standard deviations $\omega_{\psi_{L}}$ and $\omega_{\psi_{S}}$. The individual IDs of single cells classified as LT-HSC and ST-HSC and mice to which the single cells were transplanted in the single-cell transplantation are denoted as $n_{L1},...,n_{L75}$ and $n_{S1},...,n_{S39}$, respectively. Since the number of LT-HSCs and ST-HSCs in competitors is unknown and the number in transplanted BM cells is expected to be heterogeneous, their distribution follows the following formula:

(Equation 23) $$\log\left(\phi_{k}\right)=\log\left(\phi_{k,pop}\right)+\eta_{\phi ,k}\left(k=n_{L1},...,n_{L75},n_{S1},...,n_{S39},n_{B8},...,n_{B8}\right)$$

$\eta_{\phi ,k}$ is a random effect following normal distributions with mean 0 and standard deviations $\omega_{\phi ,k}$. The individual IDs of mice in the BM transplantation are denoted as $n_{B1},...,n_{B8}$.

The parameters of the model were estimated by fitting the mathematical model to the corresponding data, assuming the following observation model:

(Equation 24) $$\log\left(\frac{y_{F,i,k}\left(t\right)}{100-y_{F,i,k}\left(t\right)}\right)=\log\left(\frac{f_{F,i,k}\left(t\right)}{100-f_{F,i,k}\left(t\right)}\right)+a_{F,i}\varepsilon_{F,i,k,t}$$

(Equation 25) $$\log\left(y_{T,i,k}\left(t\right)\right)=\log\left(f_{T,i,k}\left(t\right)\right)+a_{T,i}\varepsilon_{T,i,k,t}$$

where,

(Equation 26) $$f_{F,i,k}\left(t\right)=F_{i,k}\left(t\right)(i=n_{L1},...,n_{L75},n_{S1},...,n_{S39})$$

(Equation 27) $$f_{F,i,k}\left(t\right)={\hat{F}}_{i,k}\left(t\right)\left(i=n_{B8},...,n_{B8}\right)$$

(Equation 28) $$f_{T,i,k}\left(t\right)=T_{i,k}\left(t\right)$$

$\varepsilon_{F,i,k,t}$ and $\varepsilon_{T,i,k,t}$ are observation errors for chimerism and CBC data, $y_{F,i,k}\left(t\right)$ and $y_{T,i,k}\left(t\right)$, of lineage i in individual k observed at time t, which follows a standard normal distribution. $a_{F,i}$ and $a_{T,i}$ define variances of observation errors for chimerism and CBC. To account for the data points under the detection limit (detection limit is 0.001% for chimerism), the likelihood function assumed the data under the detection limit are censored. The estimated population parameters and individual parameters are listed in Tables S1 and S2, respectively.

#### Clustering of influx and PB chimerism datasets

Hierarchical clustering was performed and figures are plotted in R (http://www.r-project.org) using the ComplexHeatmap package.^31^^,^^32^ The influx calculated by the mathematical model ((Equation 9), (Equation 10), (Equation 11), (Equation 12), (Equation 13), (Equation 14), (Equation 15))) with estimated parameters was normalized by dividing by the maximum of each influx value to range from 0 to 1. It was then normalized to have a mean of 0 and variance of 1 within individual mice. Chimerism values in PB were normalized from the original values to have a mean of 0 and variance of 1 within individuals. Hierarchical clustering was performed using complete linkage.

### Quantification and statistical analysis

P-values in Figures 1, 3, and 4 were calculated by the Student’s t test for the mean of young and aged HSCs with Bonferroni correction and significance level as 0.05.

## Supplemental information

Document S1. Figures S1–S9

Table S1. Excel file containing estimated population parameters in a nonlinear mixed-effect model, related to STAR Methods ∗95% confidence interval, estimated as 2.5 and 97.5 percentiles by the bootstrap method. ∗∗Calculated with estimated values of other parameters based on definitions or assumptions described in STAR Methods. ∗∗∗Fixed in the parameter estimation.

Table S2. Excel file containing estimated individual parameters in nonlinear mixed-effect model is too large to fit in a PDF, related to STAR Methods

## References

[1] Kiel M.J., Yilmaz O.H., Iwashita T., Yilmaz O.H., Terhorst C., Morrison S.J. (2005). SLAM family receptors distinguish hematopoietic stem and progenitor cells and reveal endothelial niches for stem cells. Cell.

[2] Morita Y., Ema H., Nakauchi H. (2010). Heterogeneity and hierarchy within the most primitive hematopoietic stem cell compartment. J. Exp. Med..

[3] Wilson N.K., Kent D.G., Buettner F., Shehata M., Macaulay I.C., Calero-Nieto F.J., Sánchez Castillo M., Oedekoven C.A., Diamanti E., Schulte R. (2015). Combined Single-Cell Functional and Gene Expression Analysis Resolves Heterogeneity within Stem Cell Populations. Cell Stem Cell.

[4] Yamamoto R., Morita Y., Ooehara J., Hamanaka S., Onodera M., Rudolph K.L., Ema H., Nakauchi H. (2013). Clonal analysis unveils self-renewing lineage-restricted progenitors generated directly from hematopoietic stem cells. Cell.

[5] Yamamoto R., Wilkinson A.C., Ooehara J., Lan X., Lai C.-Y., Nakauchi Y., Pritchard J.K., Nakauchi H. (2018). Large-Scale Clonal Analysis Resolves Aging of the Mouse Hematopoietic Stem Cell Compartment. Cell Stem Cell.

[6] Osawa M., Hanada K., Hamada H., Nakauchi H. (1996). Long-term lymphohematopoietic reconstitution by a single CD34-low/negative hematopoietic stem cell. Science.

[7] Yamamoto R., Wilkinson A.C., Nakauchi H. (2018). Changing concepts in hematopoietic stem cells. Science.

[8] Sanjuan-Pla A., Macaulay I.C., Jensen C.T., Woll P.S., Luis T.C., Mead A., Moore S., Carella C., Matsuoka S., Bouriez Jones T. (2013). Platelet-biased stem cells reside at the apex of the haematopoietic stem-cell hierarchy. Nature.

[9] Carrelha J., Meng Y., Kettyle L.M., Luis T.C., Norfo R., Alcolea V., Boukarabila H., Grasso F., Gambardella A., Grover A. (2018). Hierarchically related lineage-restricted fates of multipotent haematopoietic stem cells. Nature.

[10] Carrelha J., Mazzi S., Winroth A., Hagemann-Jensen M., Ziegenhain C., Högstrand K., Seki M., Brennan M.S., Lehander M., Wu B. (2024). Alternative platelet differentiation pathways initiated by nonhierarchically related hematopoietic stem cells. Nat. Immunol..

[11] Michor F., Hughes T.P., Iwasa Y., Branford S., Shah N.P., Sawyers C.L., Nowak M.A. (2005). Dynamics of chronic myeloid leukaemia. Nature.

[12] Bernitz J.M., Kim H.S., MacArthur B., Sieburg H., Moore K. (2016). Hematopoietic Stem Cells Count and Remember Self-Renewal Divisions. Cell.

[13] Morcos M.N.F., Li C., Munz C.M., Greco A., Dressel N., Reinhardt S., Sameith K., Dahl A., Becker N.B., Roers A. (2022). Fate mapping of hematopoietic stem cells reveals two pathways of native thrombopoiesis. Nat. Commun..

[14] Ema H., Uchinomiya K., Morita Y., Suda T., Iwasa Y. (2016). Repopulation dynamics of single haematopoietic stem cells in mouse transplantation experiments: Importance of stem cell composition in competitor cells. J. Theor. Biol..

[15] Iwanami S., Yamamoto R. (2025).

[16] Iwanami S. (2025).

[17] Busch K., Klapproth K., Barile M., Flossdorf M., Holland-Letz T., Schlenner S.M., Reth M., Höfer T., Rodewald H.-R. (2015). Fundamental properties of unperturbed haematopoiesis from stem cells in vivo. Nature.

[18] Cosgrove J., Hustin L.S.P., de Boer R.J., Perié L. (2021). Hematopoiesis in numbers. Trends Immunol..

[19] Dykstra B., Olthof S., Schreuder J., Ritsema M., de Haan G. (2011). Clonal analysis reveals multiple functional defects of aged murine hematopoietic stem cells. J. Exp. Med..

[20] Haas S., Hansson J., Klimmeck D., Loeffler D., Velten L., Uckelmann H., Wurzer S., Prendergast Á.M., Schnell A., Hexel K. (2015). Inflammation-induced emergency megakaryopoiesis driven by hematopoietic stem cell-like megakaryocyte progenitors. Cell Stem Cell.

[21] Rodriguez-Fraticelli A.E., Wolock S.L., Weinreb C.S., Panero R., Patel S.H., Jankovic M., Sun J., Calogero R.A., Klein A.M., Camargo F.D. (2018). Clonal analysis of lineage fate in native haematopoiesis. Nature.

[22] Poscablo D.M., Worthington A.K., Smith-Berdan S., Rommel M.G.E., Manso B.A., Adili R., Mok L., Reggiardo R.E., Cool T., Mogharrab R. (2024). An age-progressive platelet differentiation path from hematopoietic stem cells causes exacerbated thrombosis. Cell.

[23] Li J.-J., Liu J., Li Y.E., Chen L.V., Cheng H., Li Y., Cheng T., Wang Q.-F., Zhou B.O. (2024). Differentiation route determines the functional outputs of adult megakaryopoiesis. Immunity.

[24] Pang W.W., Price E.A., Sahoo D., Beerman I., Maloney W.J., Rossi D.J., Schrier S.L., Weissman I.L. (2011). Human bone marrow hematopoietic stem cells are increased in frequency and myeloid-biased with age. Proc. Natl. Acad. Sci. USA.

[25] Gekas C., Graf T. (2013). CD41 expression marks myeloid-biased adult hematopoietic stem cells and increases with age. Blood.

[26] Grover A., Sanjuan-Pla A., Thongjuea S., Carrelha J., Giustacchini A., Gambardella A., Macaulay I., Mancini E., Luis T.C., Mead A. (2016). Single-cell RNA sequencing reveals molecular and functional platelet bias of aged haematopoietic stem cells. Nat. Commun..

[27] Wahlestedt M., Erlandsson E., Kristiansen T., Lu R., Brakebusch C., Weissman I.L., Yuan J., Martin-Gonzalez J., Bryder D. (2017). Clonal reversal of ageing-associated stem cell lineage bias via a pluripotent intermediate. Nat. Commun..

[28] Wojtowicz E.E., Mistry J.J., Uzun V., Hellmich C., Scoones A., Chin D.W., Kettyle L.M., Grasso F., Lord A.M., Wright D.J. (2023). Panhematopoietic RNA barcoding enables kinetic measurements of nucleate and anucleate lineages and the activation of myeloid clones following acute platelet depletion. Genome Biol..

[29] Urbanus J., Cosgrove J., Beltman J.B., Elhanati Y., Moral R.A., Conrad C., van Heijst J.W., Tubeuf E., Velds A., Kok L. (2023). DRAG in situ barcoding reveals an increased number of HSPCs contributing to myelopoiesis with age. Nat. Commun..

[30] Monolix 2024R1, Simulations Plus.

[31] Gu Z., Eils R., Schlesner M. (2016). Complex heatmaps reveal patterns and correlations in multidimensional genomic data. Bioinformatics.

[32] Gu Z. (2022). Complex heatmap visualization. Imeta.

