# Supplementary material for: Stable platelet production via the bypass pathway explains long-term hematopoietic stem cell reconstitution
Source: iScience. 2025 Apr 29;28(6):112547. doi: 10.1016/j.isci.2025.112547 (PMC12144409; doi:10.1016/j.isci.2025.112547)
Supplement: Supplementary file 1 — Document S1. Figures S1–S9 [file mmc1.pdf]

iScience, Volume *28*

Supplemental information

Stable platelet production via the bypass pathway explains long-term hematopoietic stem cell reconstitution

Shoya Iwanami, Toshiko Sato, Hiroshi Haeno, Longchen Xu, Keimyo Imamura, Jun Ooehara, Xun Lan, Hiromitsu Nakauchi, Shingo Iwami, and Ryo Yamamoto

Supplementary figures

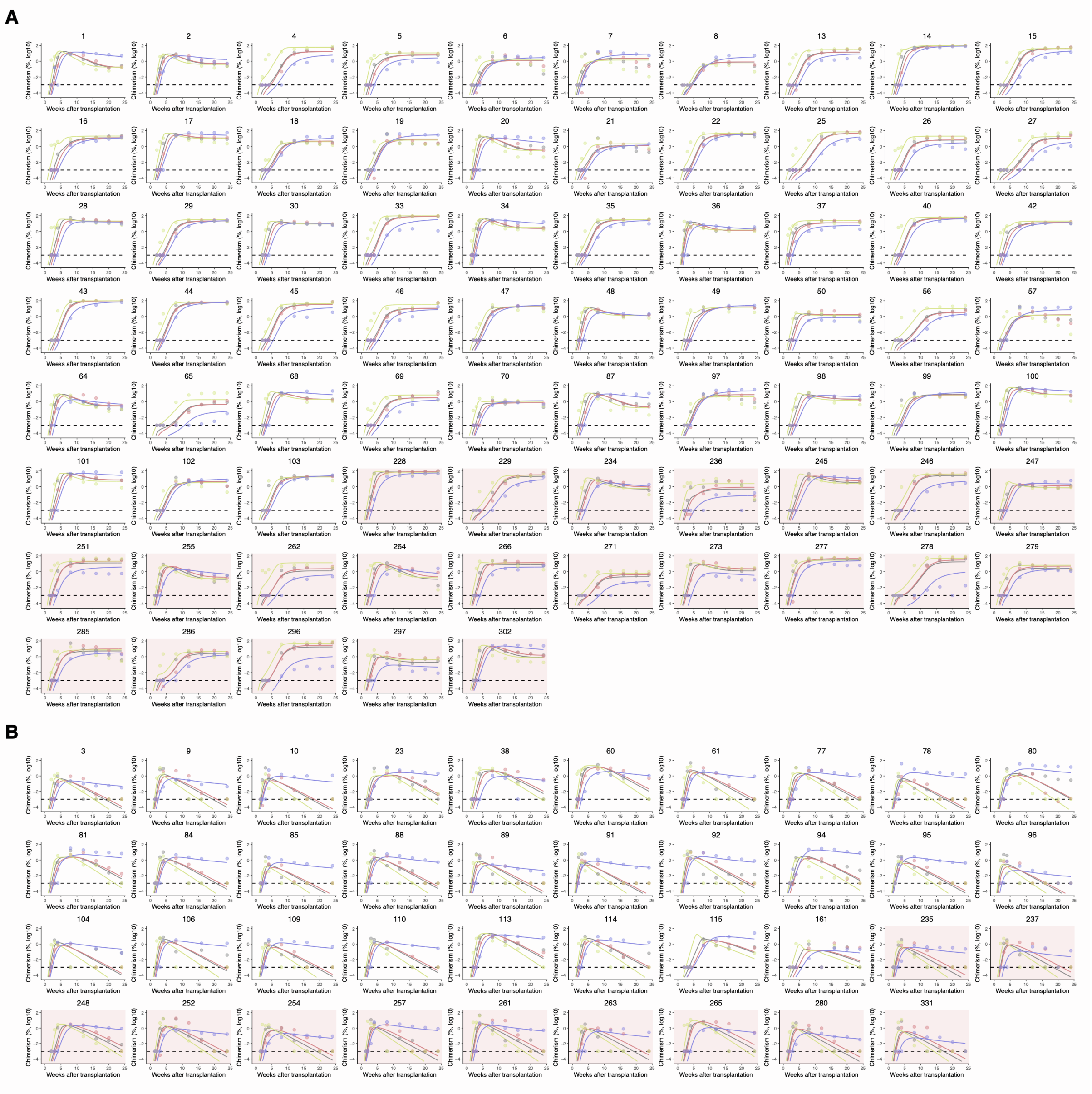

Figure S1 Individual chimerism data in the single-cell transplantation assay and best-fit model

(A-B) Individual chimerism data from the single-cell transplantation assay (dots) and expected values of the mathematical model calculated with best-fit parameters (lines). Dots and lines in gray, red, yellow, and blue correspond to neutrophils/monocytes, erythrocytes, platelets, and B cells, respectively. Panels with white and pink backgrounds are experiments in which young and aged HSCs were transplanted, respectively. **(A)** and **(B)** are experiments in which transplanted HSCs were classified as LT-HSC and ST-HSC, respectively.

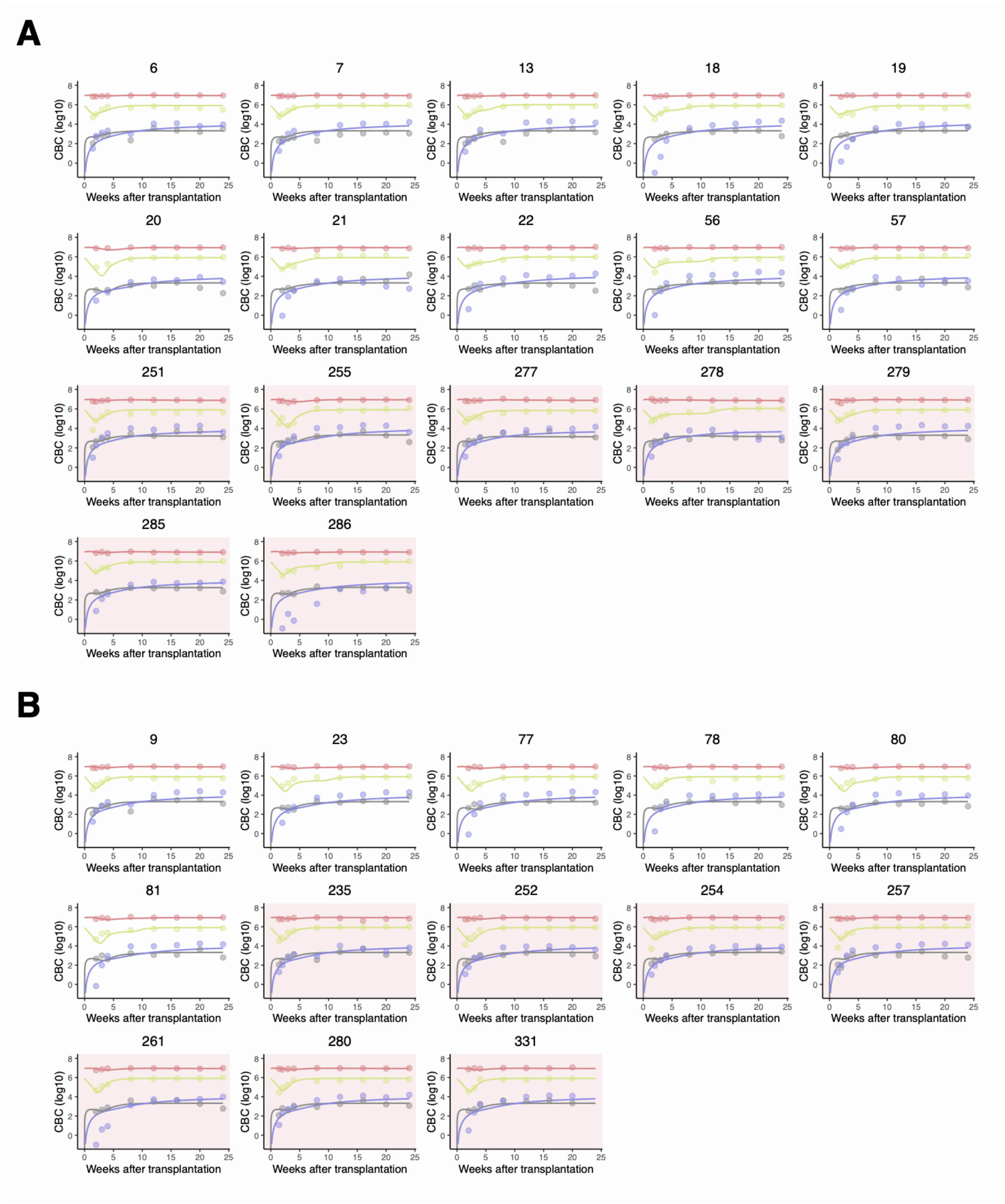

Figure S2 Individual CBC data in the single-cell transplantation assay and best-fit model

(A-B) Individual CBC data from the single-cell transplantation assay (dots) and expected values of the mathematical model calculated with best-fit parameters (lines). Dots and lines in gray, red, yellow, and blue correspond to neutrophils/monocytes, erythrocytes, platelets, and B cells, respectively. Panels with white and pink backgrounds are experiments in which young and aged HSCs were transplanted,

respectively. **(A)** and **(B)** are experiments in which transplanted HSCs were classified as LT-HSC and ST-HSC, respectively.

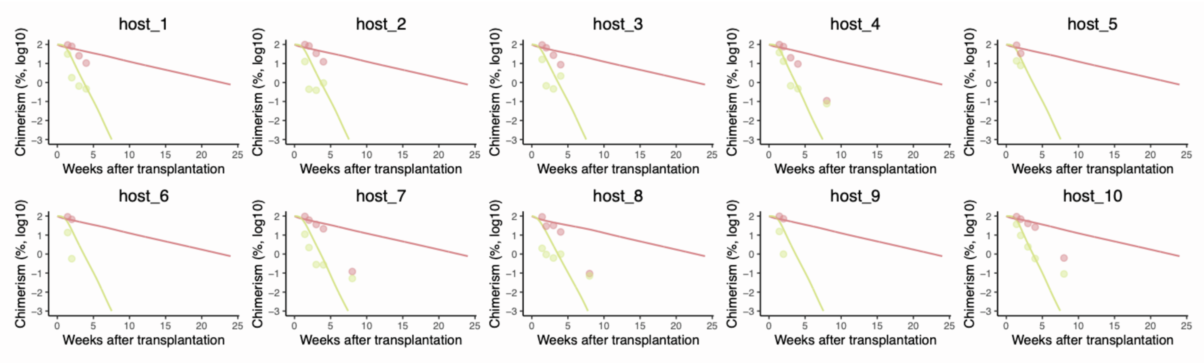

Figure S3 Individual recipient chimerism data in the bone marrow cell transplantation assay and best-fit model

Individual recipient chimerism data from the bone marrow cell transplantation assay (dots) and expected values of the mathematical model calculated with best-fit parameters (lines). Dots and lines in red and yellow correspond to erythrocytes and platelets, respectively.

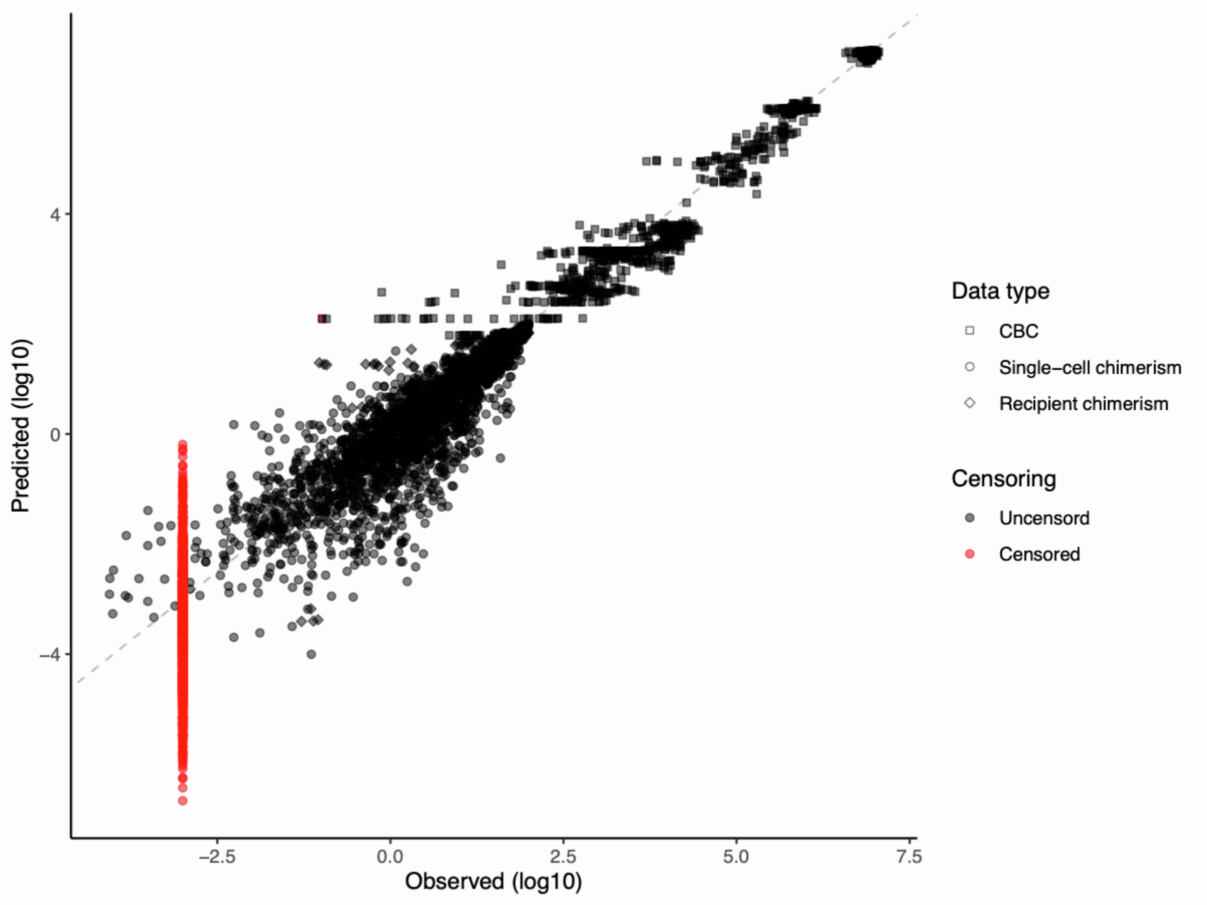

Figure S4 Comparison of observed data with individual model prediction.

Comparison of the observed data used in the data analysis (**Figures S1, S2 and S3**) with the value of the corresponding mathematical model predictions. The different types of data are indicated by the different shapes of the markers.

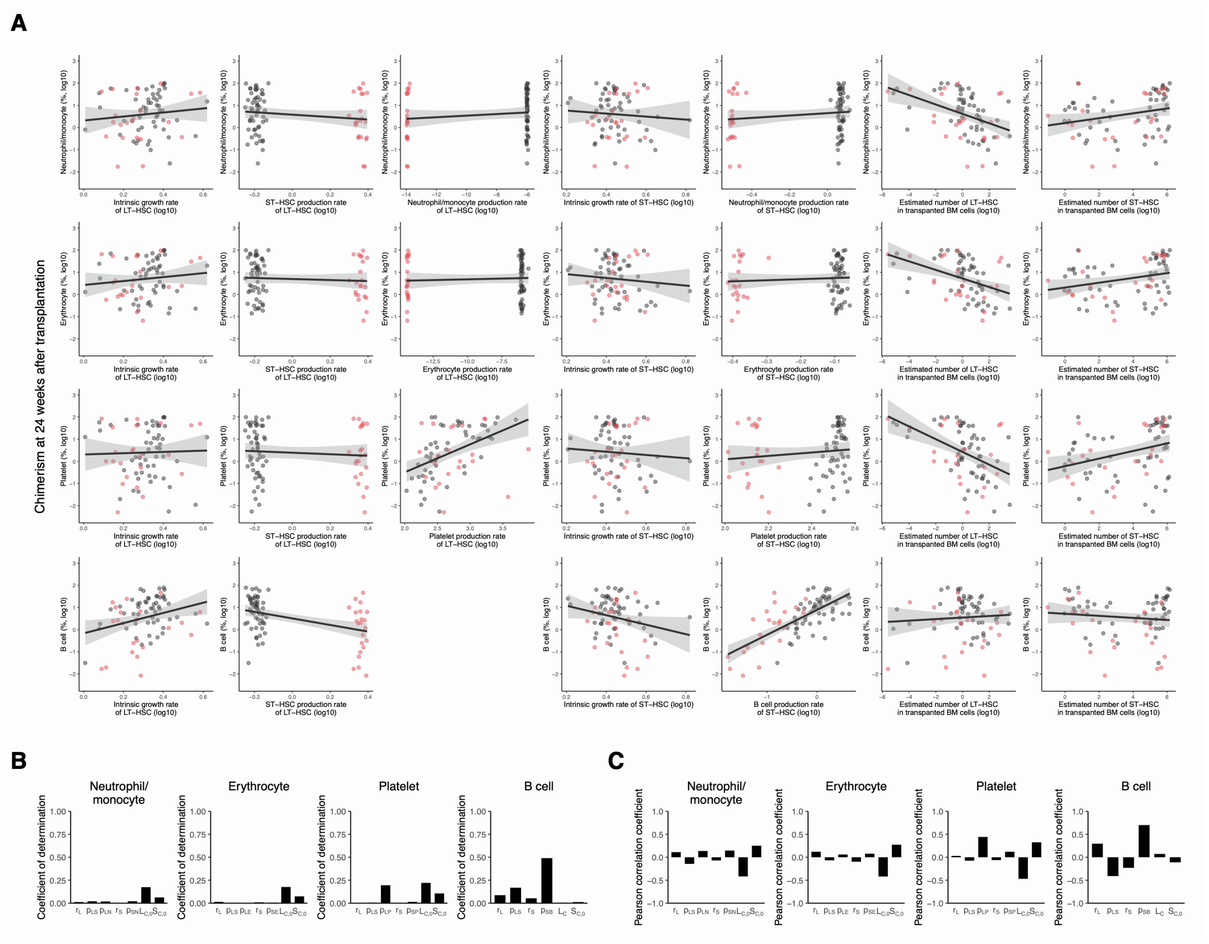

Figure S5 Comparison of estimated model parameters with experimentally obtained chimerism data

(A) Correlation between parameters in the mathematical model estimated from time-course data of chimerism in the single-cell transplantation assay and chimerism values of the relevant blood cell lines at 24 weeks after transplantation. Black and red dots correspond to the experiments in which young and aged HSCs were transplanted. Lines and shaded areas were obtained by linear regression. **(B-C)** Coefficient of determination **(B)** and Pearson correlation **(C)** coefficient in **(A)**.

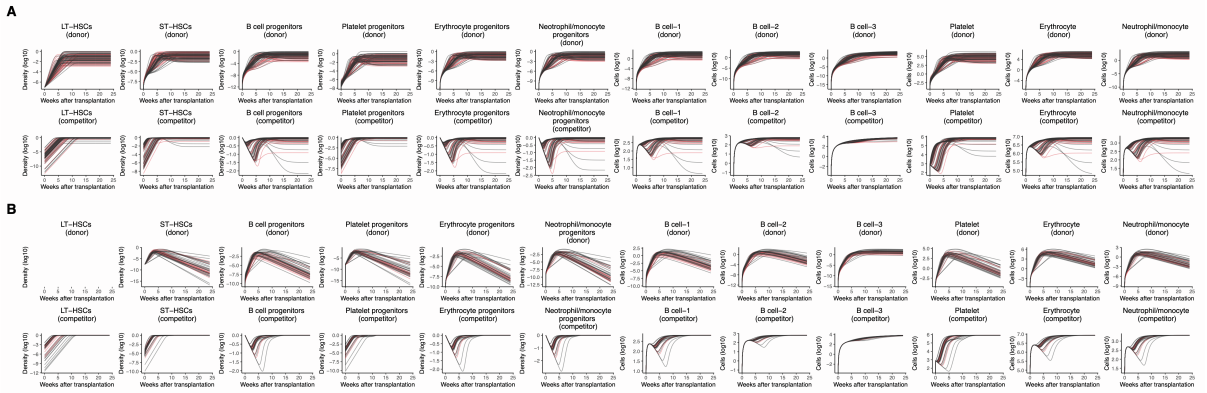

Figure S6 Expected density and number of each cell population in the single-cell transplantation assay

(A-B) Individual densities and numbers of each cell population in the single-cell transplantation of LT-HSC **(A)** and ST-HSC **(B)** calculated by the mathematical model with best-fit parameters. Black and red lines correspond to experiments in which young and aged HSCs were transplanted, respectively.

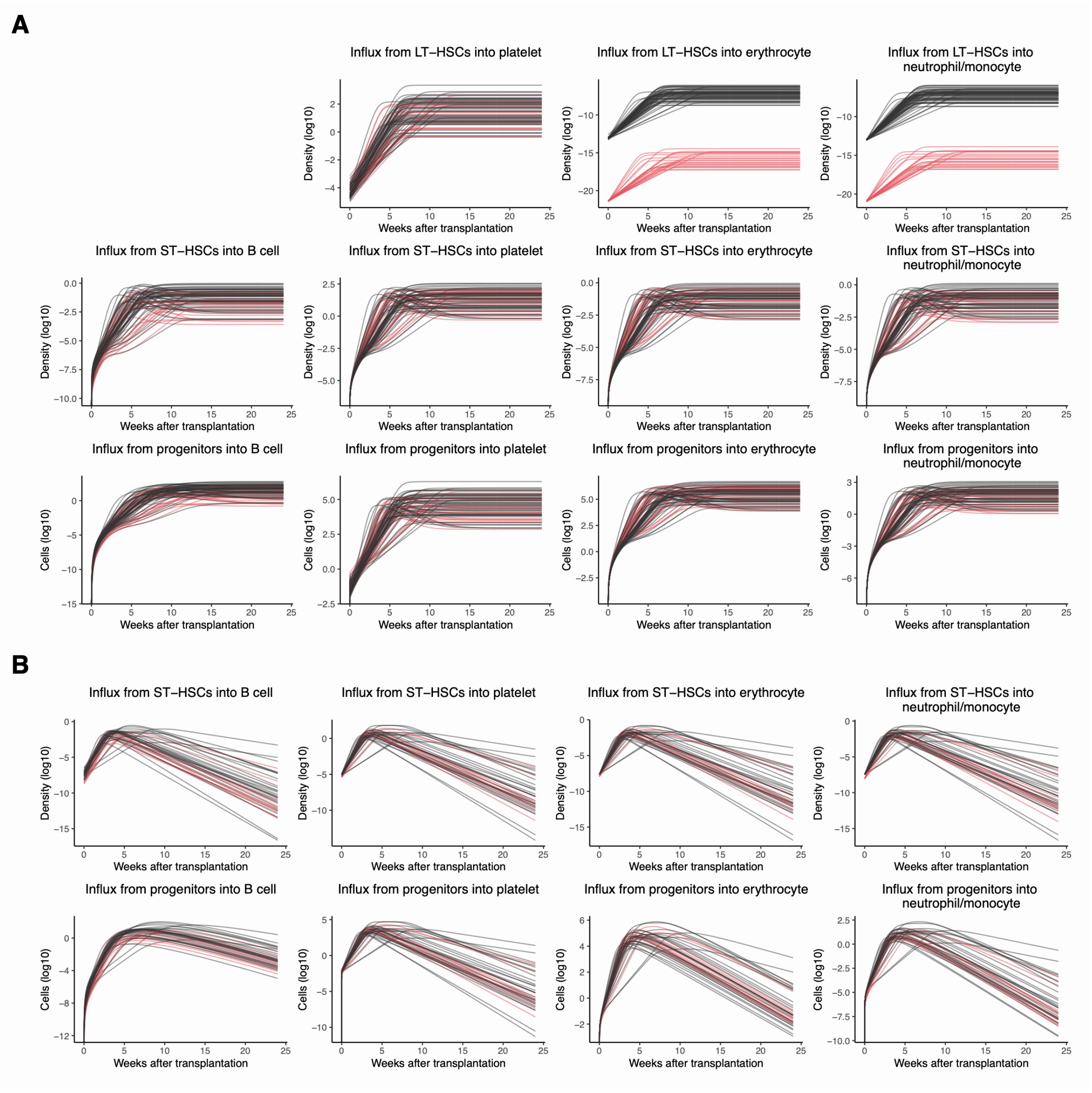

Figure S7 Expected influx of each cell population in the single-cell transplantation assay

(A-B) Production from the upper cell populations to each cell population at each time after transplantation calculated by the mathematical model with best-fit parameters (i.e., influx from LT-HSC into progenitors without going through ST-HSC, $p_{Li,D,k}L_{i,D,k}(t)$ ($i = N, E, P$, and $k = n_{L1}, \ldots, n_{L75}, n_{S1}, \ldots, n_{S39}$), influx from ST-HSC to progenitors, $p_{Si,D,k}S_{i,D,k}(t)$ ($i = N, E, P, B$ and $k = n_{L1}, \ldots, n_{L75}, n_{S1}, \ldots, n_{S39}$), and influx from progenitors into mature cells, $\alpha_i d_i Y_{i,D,k}(t)$ ($i = N, E, P$ and $k = n_{L1}, \ldots, n_{L75}, n_{S1}, \ldots, n_{S39}$) and $d_B E_{B2,D,k}(t)$ ($k = n_{L1}, \ldots, n_{L75}, n_{S1}, \ldots, n_{S39}$)). Black and red lines correspond to the experiments in which young and aged HSCs were transplanted, respectively.

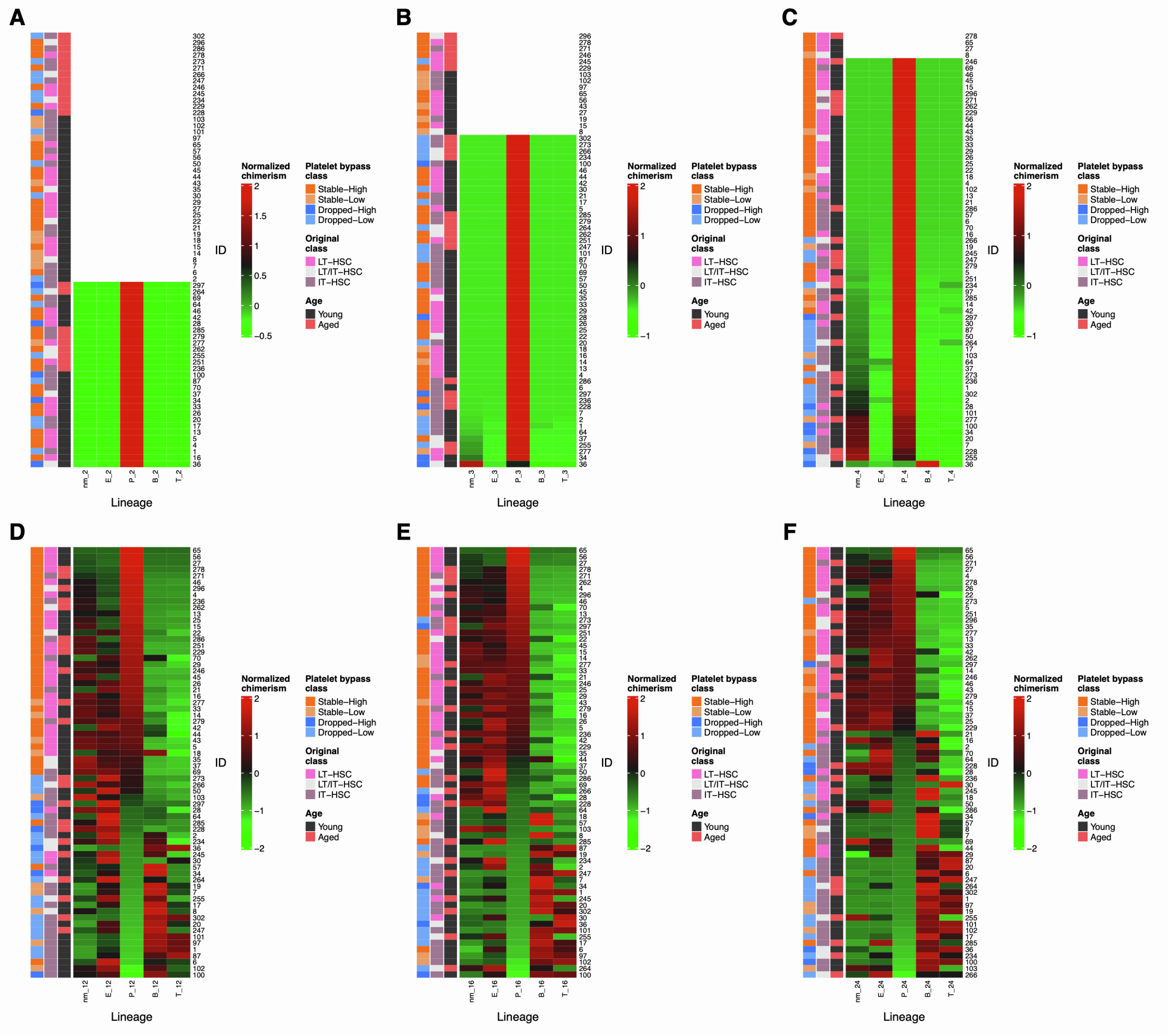

Figure S8 Normalized chimerism at 2-24 weeks after transplantation

(A-F) Normalized chimerism at 2 **(A)**, 3 **(B)**, 4 **(C)**, 12 **(D)**, 16 **(E)**, and 24 **(F)** weeks after transplantation with HSCs classified as LT-HSC or IT-HSC. The observations in which chimerism in all lineages were below the detection limit are indicated by white. The individuals are sorted in decreasing order using the normalized chimerism value of the platelet. The class defined by P-bypass, the class defined by the duration of reconstitution after transplantation, and age for transplanted HSCs are shown next to the heatmap.

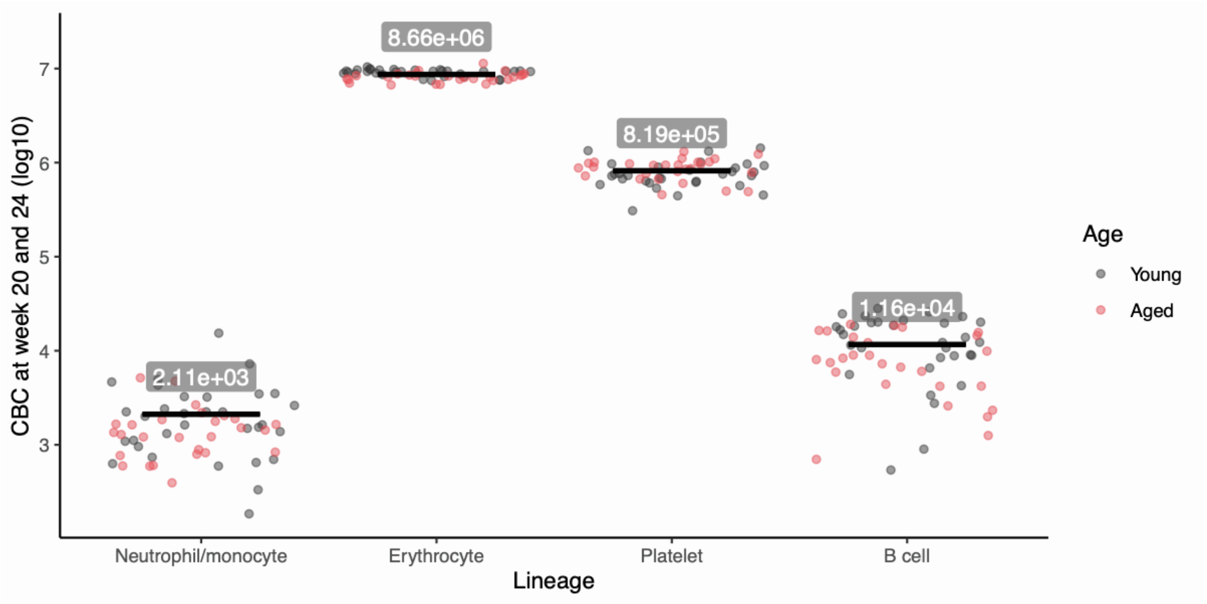

Figure S9 Measured CBC in single-cell transplantation assays

CBC values (dots) and their means (lines) for each blood cell lineage at 20 and 24 weeks after transplantation in single-cell transplantation assays in which CBCs were measured. The means were calculated by pooling the two time points and the types of young and aged HSCs.
